# Loss of CLDN5 in podocytes deregulates WIF1 to activate WNT signaling and contributes to kidney disease

**DOI:** 10.1038/s41467-022-29277-6

**Published:** 2022-03-24

**Authors:** Hui Sun, Hui Li, Jie Yan, Xiangdong Wang, Mengyuan Xu, Mingxia Wang, Baozhen Fan, Jieying Liu, Ninghua Lin, Xin Wang, Li Li, Shengtian Zhao, Yongfeng Gong

**Affiliations:** 1 Department of Physiology, Binzhou Medical University, 264003 Yantai, PR China; 2 Department of Urology, West China Hospital, Sichuan University, 610041 Chengdu, PR China; 3 Department of Anesthesiology, Binzhou Medical University, 264003 Yantai, PR China; 4 Department of Pathophysiology, Qingdao University, 266071 Qingdao, PR China; 5 Department of Urology, Binzhou Medical University Hospital, 256603 Yantai, PR China; 6 Shandong Provincial Engineering Laboratory of Urologic Tissue Reconstruction, 250021 Jinan, PR China; 7 Department of Urology, Shandong Provincial Hospital Affiliated to Shandong First Medical University, 250021 Jinan, PR China

**Keywords:** Renal fibrosis, Tight junctions, Kidney

## Abstract

Although mature podocytes lack tight junctions, tight junction integral membrane protein claudin-5 (CLDN5) is predominantly expressed on plasma membranes of podocytes under normal conditions. Using podocyte-specific Cldn5 knockout mice, we identify CLDN5 as a crucial regulator of podocyte function and reveal that Cldn5 deletion exacerbates podocyte injury and proteinuria in a diabetic nephropathy mouse model. Mechanistically, CLDN5 deletion reduces ZO1 expression and induces nuclear translocation of ZONAB, followed by transcriptional downregulation of WNT inhibitory factor-1 (WIF1) expression, which leads to activation of WNT signaling pathway. Podocyte-derived WIF1 also plays paracrine roles in tubular epithelial cells, as evidenced by the finding that animals with podocyte-specific deletion of Cldn5 or Wif1 have worse kidney fibrosis after unilateral ureteral obstruction than littermate controls. Systemic delivery of WIF1 suppresses the progression of diabetic nephropathy and ureteral obstruction-induced renal fibrosis. These findings establish a function for podocyte CLDN5 in restricting WNT signaling in kidney.

## Abstract (web summary)

Claudin-5 is a tight junction integral membrane protein, but it is also expressed in mature podocytes which lack tight junctions. Here the authors report that podocyte claudin-5 regulates WNT signaling activity by modulating WIF1 expression, and its downregulation contributes to kidney disease progression in mice.

## Introduction

Podocyte injury is now appreciated to be at the crux of many forms of proteinuric kidney diseases^1^, and the identification of novel pathophysiological pathways and molecules in podocytes is essential for the prevention of glomerular disease progression and the discovery of new avenues for treatment. During development, tight junctions (TJs) connect immature podocytes at an early stage and disappear along with the widening of the intercellular spaces and the appearance of slit diaphragms (SDs)^2,3^. Although mature podocytes lack TJs and form SDs between opposing foot processes (FPs), several claudins (CLDNs), which are the TJ-specific integral membrane protein, such as CLDN3 in nephrin (NPHS1) knockout mice^4^ and CLDN1 in animals with diabetic nephropathy (DN)^5^, have been detected in podocytes of adult mouse kidneys. However, CLDN5 is the major CLDN expressed on the plasma membranes of mature podocytes under normal conditions^6^. Surprisingly, even 10 years after the discovery of CLDN5 in podocytes, little is known about the functional role of CLDN5 in glomerular physiology and disease development.

CLDN5 is unique among CLDN family members as its predominant expression in the endothelium and non-redundant function in the control of vascular permeability. CLDN5-deficient mice are born alive, but die within 10 h after birth without any morphological abnormalities^7^. In the current study, using podocyte-specific Cldn5 knockout mice as a model, we report a previously unknown role for CLDN5 in the regulation of podocyte homeostasis. We reveal a mechanism whereby CLDN5 absence affected podocyte disease states through the transcriptional downregulation of WNT inhibitor factor-1 (WIF1) expression, which led to activation of the WNT signaling pathway. In contrast, knockout of Wif1 in podocytes resulted in the development of proteinuria and the typical ultrastructural changes observed in podocyte-specific Cldn5 knockout mice. More importantly, targeted delivery of Wif1 to podocytes prevented the development of glomerular nephropathy in podocyte-specific Cldn5 knockout mice with diabetes. In mice harboring podocyte-specific deletion of Wif1, diabetes-induced proteinuria and podocyte injury were substantially exacerbated. Because WIF1 is a secreted antagonist of the WNT pathway, we hypothesized that reduced secretion of WIF1 by podocytes would result in activation of WNT signaling in renal tubule epithelial cells and lead to increased maladaptive repair of the kidney following injury. To test this hypothesis, we subjected these mice to tubular injury by introducing unilateral ureteral obstruction (UUO). We found that the animals with podocyte deletion of Cldn5 or Wif1 gene had worse kidney fibrosis following UUO when compared with littermate controls with intact podocyte WIF1 expression. Systemic delivery of WIF1 could attenuate DN and UUO-induced renal fibrosis, introducing WIF1 as a therapeutic target for mitigating kidney injury. We also provide mechanistic insights into the regulation of Wif1 by CDLN5 by showing that CLDN5 affected the subcellular localization of the transcription factor ZO1 associated nucleic acid-binding protein (ZONAB), which directly regulated Wif1 expression through the interaction with its 3′-UTR.

## Results

### Generation and characterization of podocyte-specific Cldn5 knockout mice

**Fig. 1:**
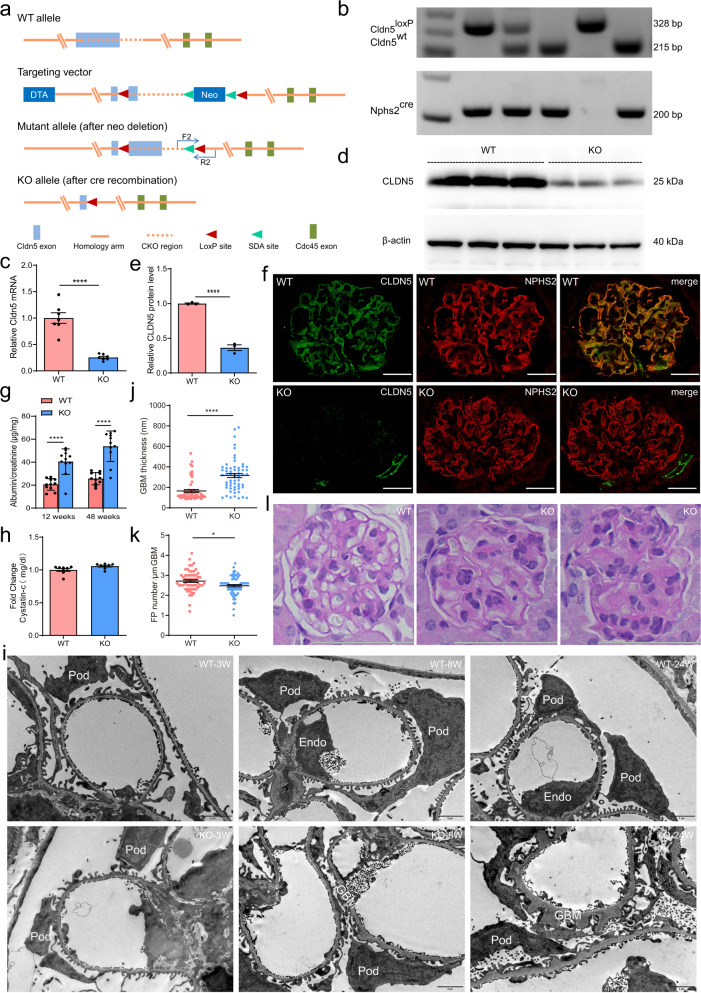
Generation and characterization of podocyte-specific Cldn5 knockout mice. **a** Gene targeting strategy. The diagram showing WT (wild-type) Cldn5 locus, targeting construct, mutant allele after neo deletion, and KO (knockout) allele after Cre recombination. **b** Genotyping of offsprings by PCR showing WT (215 bp), heterozygous (215 bp and 328 bp), and homozygous (328 bp) alleles. The Nphs2 Cre recombinase transgene was identified as a 200 bp PCR product. **c** qRT-PCR analysis of isolated glomerulus from Cldn5^ctrl^ (WT) and Cldn5^podKO^ (Cldn5 KO) mice showing 75% reduction in Cldn5 mRNA expression in the KO mice (*n* = 7 biologically independent animals, ****P < 0.0001). **d** Western blotting analysis of isolated glomerulus confirmed the deletion of CLDN5 protein in the KO mice. **e** Bar graphs showing the mean ± SEM of the fold change in CLDN5 expression determined by densitometric analysis (*n* = 3 biologically independent animals, ****P < 0.0001). **f** CLDN5 and NPHS2 immunostaining of kidney sections from WT and Cldn5 KO mice. Scale bars, 20 μm. **g** Cldn5 KO mice displayed a significant increase in albumin levels, as determined by ELISA, at 12 and 48 weeks of age (*n* = 11 biologically independent animals, ****P < 0.0001). The *y* axis shows the ratio of albumin to creatinine in spot urine compared to that in the control group. **h** Graph of plasma cystatin C levels in KO mice compared to control littermates at 8 weeks of age (*n* = 8 biologically independent animals). **i**–**k** TEM analysis of WT and Cldn5 KO mice at 3, 8, and 24 weeks of age (**i**) and quantification of GBM thickness (**j**) and FP number (**k**) at 24 weeks of age (*n* = 3 biologically independent animals and 20 images per group, *P < 0.05, ****P < 0.0001). Scale bars, 5 μm. **l** PAS-stained images of kidney cortex in WT and Cldn5 KO mice showing thickening of GBM, mesangial expansion, and glomerular matrix accumulation in KO mice at 24 weeks. Scale bars, 100 μm. Data are presented as mean values ± SEM. Two-tailed unpaired Student’s *t* test was used for statistical comparisons (**c**, **e**, **g**, **h**, **j**, **k**). Source data are provided as a Source data file.

To bypass the postnatal lethality of constitutive deletion of CLDN5 and investigate the role of CLDN5 specifically in podocytes, we created Cldn5^loxP^ mice, in which the exon 1 of the Cldn5 mutant allele is flanked by loxP sites, on the C57BL/6J background (Fig. 1a). Next, we generated mice with podocyte-specific deletion of Cldn5 by intercrossing Nphs2^cre^ and Cldn5^loxP/loxP^ animals (Fig. 1b). Because Cre is known to have nonspecific effects that could influence podocytes^8^, we studied 2 mouse lines: Nphs2-Cre^+/−^/Cldn5^loxP/+^ mice and Nphs2-Cre^+/−^/Cldn5^loxP/loxP^ mice, hereafter referred to as Cldn5^ctrl^ mice and Cldn5^podKO^ mice, respectively. Successful deletion of Cldn5 in Cldn5^podKO^ mice was confirmed by performing relative quantitative real-time PCR (qRT-PCR) using isolated glomerulus (Fig. 1c). Likewise, western blotting analyses of CLDN5 expression showed a significant decrease in CLDN5 expression (~77%) in glomerulus from Cldn5^podKO^ mice (Fig. 1d, e), suggesting that CLDN5 expression in podocytes accounts for the majority of CLDN5 in normal glomerulus. CLDN5 co-localized with the podocyte-specific marker podocin (NPHS2) in Cldn5^ctrl^ mice, consistent with a podocyte source (Fig. 1f). Immunofluorescence staining, as indicated by the lack of CLDN5 colocalization with NPHS2 in Cldn5^podKO^ kidneys but appropriate signal in the endothelial cells of arteriole, confirmed that the Nphs2-Cre–mediated Cldn5 deletion was largely confined to podocytes (Fig. 1f). The knockout mice did not show compensated or increased expression of other TJ proteins CLDN1, CLDN3, and CLDN6 in podocytes (Supplementary Fig. 1).

We next investigated whether reduced CLDN5 expression alone could cause proteinuria directly using our engineered mice with podocyte-specific deletion of Cldn5. Cldn5^podKO^ mice showed no albuminuria in early stages, but albuminuria began to occur at around 12 weeks and continued to rise through the end of the study at 48 weeks (Fig. 1g). There was no significant difference in body weight, urine volume, or urinary osmolality throughout the observation period from 3 to 48 weeks (Supplementary Table 1). Blood cystatin C levels were within the normal ranges in both the groups (Fig. 1h). To investigate whether the appeared albuminuria was due to damage to the glomerular filtration barrier, we examined the ultrastructure of Cldn5^podKO^ and Cldn5^ctrl^ mouse kidneys by transmission electron microscopy (TEM). TEM revealed global thickening of the glomerular basement membrane (GBM) in Cldn5^podKO^ mice (Fig. 1i). GBM abnormalities were obvious at 8 weeks of age and gradually aggravated by 24 weeks (Fig. 1j). In addition, podocyte FPs appeared abnormal with broadening and effacement, which were notable in areas of severe GBM thickening (Fig. 1k). Control littermates developed mild GBM and FP changes after 24 weeks of age (Fig. 1i). Histological staining with Periodic Acid-Schiff staining (PAS) identified that Cldn5^podKO^ mice showed mesangial expansion and glomerular matrix accumulation at 24 weeks of age compared with their littermate control mice (Fig. 1l). qRT-PCR and immunofluorescence staining revealed that the expression of podocalyxin (PODXL) was reduced in Cldn5^podKO^ mice, which further confirmed the presence of podocyte damage (Supplementary Fig. 2). In summary, mice with podocyte-specific CLDN5 deficiency showed early GBM alterations followed by the development of albuminuria.

### Cldn5 deletion in podocytes accelerates DN progression

**Fig. 2:**
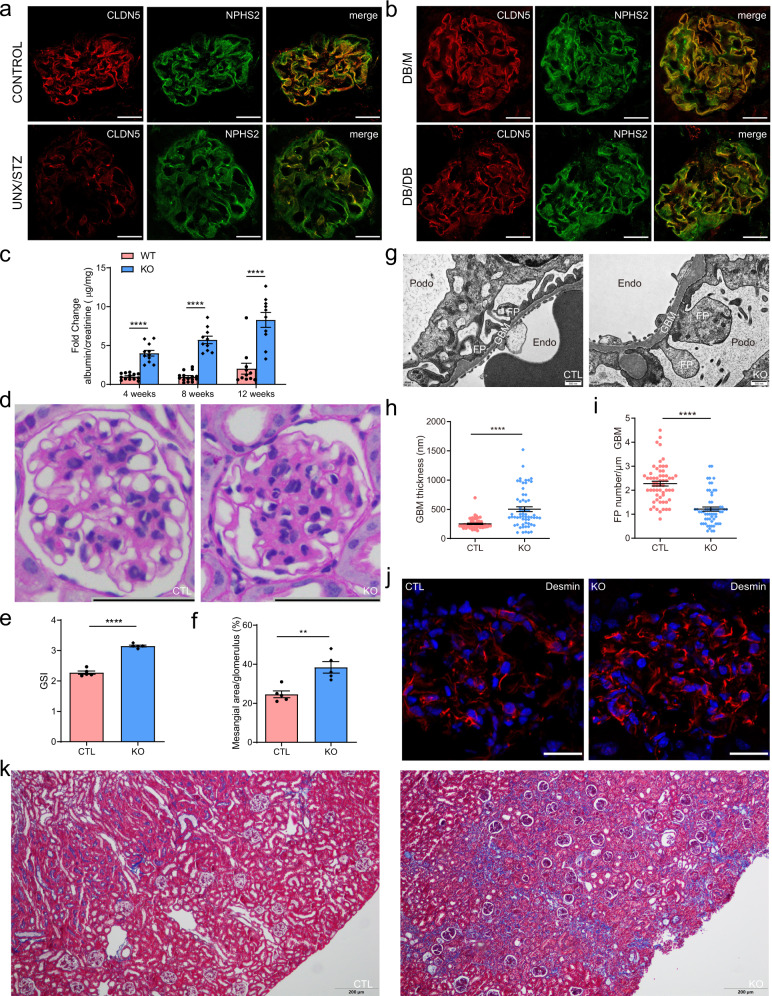
Podocyte-specific Cldn5 deletion mice show increased susceptibility to diabetic kidney injury. **a** Double immunostaining of CLDN5 and NPHS2 of kidney sections from control and DN (UNX/STZ) mice 12 weeks after the last injection of STZ. Scale bars, 20 μm. **b** Double immunostaining of CLDN5 and NPHS2 in kidney sections from control (DB/M) and DN (DB/DB) mice at 24 weeks of age. Scale bars, 20 μm. **c** Development of albuminuria (shown as the fold change in the ratio of albumin to creatinine) in CTL (WT) and KO (Cldn5 KO) mice after 4, 8, and 12 weeks of the last injection of STZ (*n* = 11 biologically independent animals, ****P < 0.0001). **d**–**f** PAS staining (**d**), GSI (**e**), and mesangial area expansion (**f**) in WT and Cldn5 KO mice 12 weeks after the last dose of STZ injection (20 glomeruli per mouse were analyzed, *n* = 5 mice per group, **P < 0.01, ****P < 0.0001). Scale bar, 50 μm. **g**–**i** TEM images of glomerular capillary loops of WT and Cldn5 KO mice (**g**) and quantification of GBM thickness (**h**) and FP number (**i**) 12 weeks after the last dose of STZ injection (*n* = 3 biologically independent animals and 20 images per group, ****P < 0.0001). Scale bar, 0.5 μm. **j** Immunostaining of podocyte injury marker desmin in kidney sections from WT and Cldn5 KO mice 12 weeks after the last STZ injection. Nuclei were visualized by DAPI. Scale bars, 20 μm. **k** MTS images of whole kidney showing interstitial fibrosis in Cldn5 KO diabetic mice 12 weeks after the last injection of STZ. Scale bars, 200 μm. Data are presented as mean values ± SEM. Two-tailed unpaired Student’s *t* test was used for statistical comparisons (**c**, **e**, **f**, **h**, **i**). Source data are provided as a Source data file.

To determine whether CLDN5 has a role in diabetic kidney disease, we first studied the expression of CLDN5 in two mouse models of DN, the unilateral nephrectomy (UNX) combined with streptozotocin (STZ)-induced type I diabetic mice and DB/DB type 2 diabetic mice, by double immunostaining for CLDN5 and NPHS2. In both strains, we found that the expression of CLDN5 was decreased, which was accompanied by an attenuation in NPHS1, ZO1, and NPHS2 expression (Fig. 2a, b, Supplementary Fig. 3a, b). To determine whether CLDN5 expression is also altered in human glomerular diseases, we queried the published transcriptomic datasets in kidney disease compiled in the Nephroseq database (nephroseq.org). CLDN5 mRNA expression was significantly reduced in the glomerulus of DN patients compared with those of healthy controls (Supplementary Fig. 3c). Then, to further investigate the effects of CLDN5 on DN development, STZ-induced DN mice with or without CLDN5 knockout were used. Cldn5^podKO^ mice did not differ in fasting blood glucose levels, HbA1c levels, food intake, urine output, or body weight compared to littermate mice in STZ-induced diabetic conditions (Supplementary Table 2). The diabetic Cldn5^podKO^ mice showed an increase in albuminuria as early 4 weeks after STZ injection, remaining elevated up to 12 weeks and reaching a difference of more than 4-fold compared with diabetic control mice of the same age (Fig. 2c). Twelve weeks after diabetes induction, glomerulosclerosis and the mesangial area were significantly increased in diabetic Cldn5^podKO^ mice when compared to their controls (Fig. 2d–f). TEM analysis demonstrated that GBM thickening and FP effacement were induced, and these effects were significantly aggravated in Cldn5^podKO^ mice post to STZ treatment (Fig. 2g–i), which is consistent with the facts that these mice had more severe albuminuria. Podocyte injury was confirmed by increased expression of the podocyte injury indicator desmin (Fig. 2j) and reduced expression of the key podocyte markers, NPHS1, NPHS2, and PODXL (Supplementary Fig. 3d–i) in the diabetic Cldn5^podKO^ mice, as compared with the diabetic Cldn5^ctrl^ group. Masson Trichrome staining (MTS) also showed a significant increase in interstitial fibrosis in the diabetic Cldn5^podKO^ mice (Fig. 2k). These results indicate that Cldn5^podKO^ mice are more susceptible to diabetic injury.

### Decreased Wif1 expression is observed in the glomerulus of podocyte-specific Cldn5 knockout mice

**Fig. 3:**
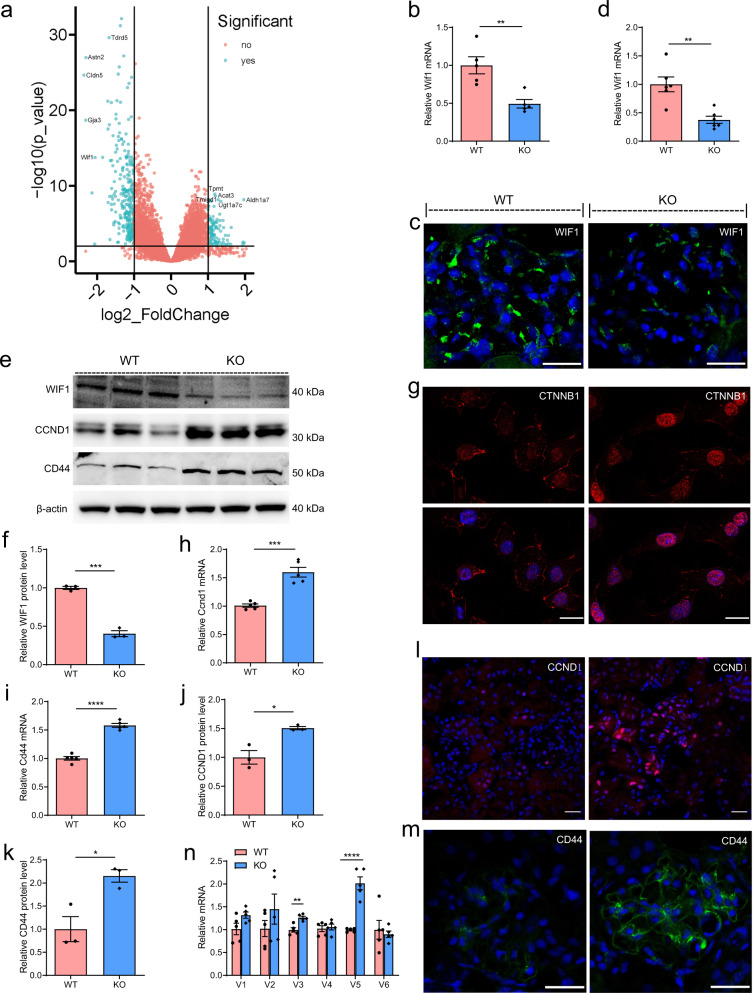
CLDN5 regulates WNT signaling pathway activity in podocytes through control the transcriptional expression of Wif1. **a** Differentially expressed genes (Cldn5 KO *VS* WT) are illustrated in the volcano plot. Differential expression analysis of two groups was performed using the DESeq2 R package (1.16.1). Genes with an adjusted *P*-value <0.05 and a fold-change greater than 2.0 were assigned as differentially expressed (*n* = 5 biologically independent animals). **b** Expression of Wif1 mRNA assessed by qRT–PCR in kidneys from WT and Cldn5 KO mice (*n* = 5 biologically independent animals, **P < 0.01). **c** Representative images of immunofluorescence for WIF1 in kidney sections from WT and Cldn5 KO mice. **d** Expression of Wif1 mRNA assessed by qRT–PCR in glomerulus from WT and Cldn5 KO mice (*n* = 6 biologically independent animals, **P < 0.01). **e** Immunoblotting for WIF1 and WNT target genes (CD44 and CCND1) in isolated glomerulus from WT and Cldn5 KO mice. **f** Bar graphs represent the mean ± SEM of fold changes corresponding to densitometric analyses for WIF1 (*n* = 3 biologically independent animals mice). ***P < 0.001 compared with glomerulus from WT mice. **g** Representative images of immunofluorescence for CTNNB1 in FACS-sorted podocytes from WT and Cldn5 KO mice. **h**, **i** mRNA expression of Ccnd1 (**h**) and Cd44 (**i**) assessed by qRT–PCR in isolated glomerulus from WT and Cldn5 KO mice (*n* = 5 biologically independent animals, ***P < 0.001, ****P < 0.0001). **j**, **k** Bar graphs represent the mean ± SEM of fold changes corresponding to densitometric analyses for CCND1 (**j**) and CD44 (**k**) (*n* = 3 biologically independent animals). *P < 0.05 compared with glomerulus from WT mice. **l**, **m** Immunofluorescence of CCND1 (**l**) and CD44 (**m**) in kidney sections from WT and Cldn5 KO mice. **n** mRNA expression of different isoforms of Cd44 assessed by qRT–PCR in isolated glomerulus from WT and Cldn5 KO mice (*n* = 5 biologically independent animals, **P < 0.01, ****P < 0.0001). Nuclei were visualized by DAPI. Scale bars, 20 μm. Data are presented as mean values ± SEM. Two-tailed unpaired Student’s *t* test was used for statistical comparisons (**b**, **d**, **f**, **h**–**k**, **n**). Source data are provided as a Source data file.

To obtain insights into what might explain the phenotype of Cldn5 deletion in podocytes, we performed RNA-seq of glomerular lysates from Cldn5^ctrl^ and Cldn5^podKO^ mice. This unbiased analysis identified 280 downregulated genes and 102 upregulated genes (Fig. 3a). We found that, among the significantly altered genes by CLDN5 deletion, change of Wif1 reached remarkably high level (Fig. 3a). The data were validated by qRT-PCR (Fig. 3b) and immunofluorescence (Fig. 3c) analysis of kidney samples. Comparable results were obtained by qRT-PCR (Fig. 3d) and western blotting analysis (Fig. 3e, f) of glomerulus isolated from mutant and control littermates. WIF1 is a secreted WNT inhibitor, that exerts its inhibitory effect on WNT signaling by binding and inhibiting the activity of extracellular WNT ligands. This finding prompted us to speculate that CLDN5 depletion may lead to downregulation of WIF1 expression, thereby activating WNT signaling. We found that WNT/β-catenin signaling was hyperactivated in Cldn5^podKO^ podocytes based upon upregulated active nuclear CTNNB1 expression in Cldn5^podKO^ podocytes compared to Cldn5^ctrl^ podocytes (Fig. 3g). Notably, we found the same expression fingerprint of CTNNB1 target genes, including increased expression in CCND1 (Fig. 3e, h, j, l) and CD44 (Fig. 3e, i, k, m) determined via qRT-PCR, western blotting, and immunofluorescence analysis. Previous studies in animals and humans have shown that CD44 is not expressed in healthy kidney, and activated parietal epithelial cells, but not podocytes, upregulate their de novo expression of CD44 during glomerular diseases^9,10^. In our study, although several CD44 isoforms were detected, the mRNA levels of CD44v3 and CD44v5 appeared to be considerably higher in glomerulus from knockout mice than in those from control littermates (Fig. 3n). Collectively, these results indicate that the WNT signaling pathway is activated by WIF1 inhibition in mutant podocytes, resulting in subsequent podocyte injury. These results identify CLDN5 as a previously undescribed regulator of WNT/β-catenin signaling activity in podocytes.

### Wif1 ablation mimics the phenotypes observed in Cldn5-deficient podocytes

**Fig. 4:**
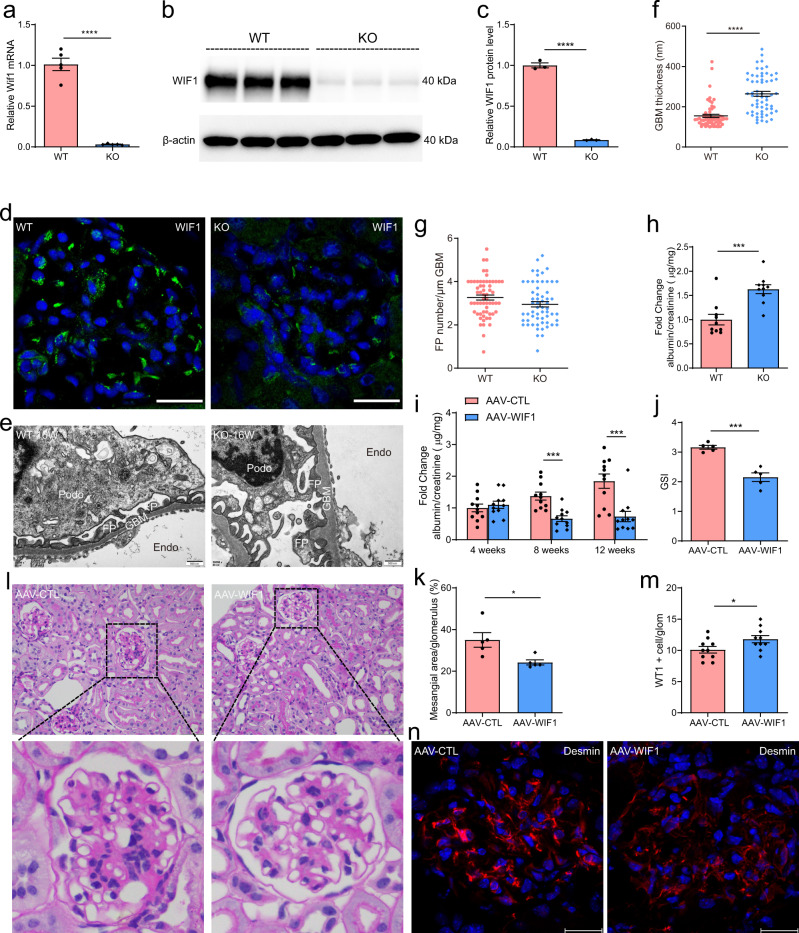
Podocyte specific Wif1 knockout (a-h) and overexpression (i-n) mice analysis. **a** qRT-PCR analysis in isolated glomerulus showing loss of Wif1 gene expression in Wif1 KO mice (*n* = 5 biologically independent animals, ****P < 0.0001). **b** Western blotting analysis for WIF1 of isolated glomerulus from WT and Wif1 KO mice. **c** Bar graphs represent the mean ± SEM of fold changes corresponding to densitometric analyses for WIF1 (*n* = 3 biologically independent animals). ****P < 0.0001 compared with kidneys from WT mice. **d** Immunofluorescence staining for WIF1 performed in WT and Wif1 KO mice. Nuclei were visualized by DAPI. Scale bar, 20 μm. **e**–**g** TEM images of glomerular capillary loops of WT and Wif1 KO mice (**e**) and quantification of GBM thickness (**f**) and FP numbers (**g**) at 16 weeks of age (*n* = 3 biologically independent animals and 20 images per group, ****P < 0.0001) at 16 weeks of age. Scale bar, 0.5 μm. **h** Albumin-to-creatinine ratios (μg/mg) of spot urine samples of 16-week-old WT and Wif1 KO mice (*n* = 10 biologically independent animals, ***P < 0.001). **i** Albumin/creatinine ratio (μg/mg) in spot urine of AAV-CTL and AAV-Wif1 treated Cldn5^podKO^ mice 4, 8, and 12 weeks after the last dose of STZ injection (*n* = 11 biologically independent animals, ***P < 0.001). **j**–**l** GSI (**j**), mesangial area expansion (**k**), and PAS staining (**l**) in AAV-CTL and AAV-WIF1 treated Cldn5^podKO^ mice 12 weeks after the last dose of STZ injection (*n* = 5 biologically independent animals, *P < 0.05, ***P < 0.001). Scale bar, 50 μm. **m** Quantification of WT1-positive cells in the AAV-CTL and AAV-WIF1 treated Cldn5^podKO^ mouse glomerulus (20 glomeruli per mouse were analyzed, *n* = 10 biologically independent animals, *P < 0.05). **n** Immunostaining of podocyte injury marker desmin of kidney sections from AAV-CTL and AAV-WIF1 treated Cldn5^podKO^ mice. Nuclei were visualized by DAPI. Scale bars, 20 μm. Data are presented as mean values ± SEM. Two-tailed unpaired Student’s *t* test was used for statistical comparisons (**a**, **c**, **f**–**k**, **m**). Source data are provided as a Source data file.

To mimic the downregulation of Wif1 expression observed in Cldn5^podKO^ mice, we next constructed conditional knockout mice with podocyte-specific ablation of Wif1 by using the Cre-LoxP system. We generated Wif1^loxP^ mice, in which the Wif1 mutated allele contains exon 3 is flanked by loxP sites, on the C57BL/6J background (Supplementary Fig. 4a). Next, we generated mice with podocyte-specific deletion of Wif1 by intercrossing Nphs2^cre^ and Wif1^loxP/loxP^ animals (Supplementary Fig. 4b). No residual Wif1 transcript or protein was detectable as determined by qRT-PCR, immunoblotting, or immunofluorescence in glomerular lysates or kidney sections in Nphs2-Cre^+/−^/Wif1^loxP/loxP^ (referred to as Wif1^podKO^) mice (Fig. 4a–d), indicating that WIF1 was expressed predominantly in podocytes in the kidney. Our data are in agreement with single-cell RNA-sequencing datasets of the mouse kidney, which indicated that Wif1 was expressed exclusively in podocytes (Supplementary Fig. 4c, d)^11,12^. To determine whether deletion of Wif1 in podocytes leads to activation of canonical WNT signaling, we studied the expression of several putative WNT/β-catenin target genes in the glomerulus. We found that CCND1 and CD44 expression was upregulated in the Wif1^podKO^ mouse glomerulus (Supplementary Fig. 4e–g). Wif1^podKO^ mice had normal renal histology at 16 weeks of age, but TEM revealed a thicker GBM that remained completely covered by FPs of podocytes, however, in areas with GBM thickenings, FP effacement was observed (Fig. 4e–g). Although genetic deletion of Wif1 resulted in a similar glomerular phenotype, the phenotypes observed in these mouse models were less severe than those in the Cldn5^podKO^ mice. Consistent with this, podocyte-specific Wif1 mutant mice developed mild albuminuria at 16 weeks of age, showing an albumin level that was 1.7-fold higher than that in control mice (Fig. 4h). The finding that Wif1^podKO^ mice did not completely phenocopy Cldn5^podKO^ mice led us to conclude that additional pathways maybe involved in kidney pathogenesis associated with Cldn5 deletion.

### AAV9-mediated WIF1 overexpression in podocytes ameliorates the progression of DN in podocyte-specific Cldn5 KO mice

To further investigate the relevance of WIF1 to the glomerular phenotype of Cldn5^podKO^ mice, we assessed whether podocyte-specific WIF1 overexpression could rescue the phenotype of Cldn5^podKO^ mice. To overexpress WIF1 in podocytes, we used an AAV9 system with kidney in situ injection^13^. Podocyte-specific WIF1 delivery rescued the glomerular injury phenotype of diabetic Cldn5^podKO^ mice, markedly decreasing urine albumin-to-creatinine ratio (Fig. 4i), reducing the glomerulosclerotic index (GSI) (Fig. 4j), and decreasing mesangial area expansion (Fig. 4k), as evidenced by PAS staining (Fig. 4l). We also observed significantly less podocyte loss in WIF1-treated group than in the mutant mice treated with control AAV (podocyte number/glomerulus: AAV-WIF1 versus AAV-CTL: 11.583 ± 0.045 versus 10.167 ± 0.035, *P* < 0.05, *n* = 10 mice/group) (Fig. 4i). Taken together, these results show striking normalization of podocytes in diabetic Cldn5^podKO^ mouse model upon WIF1 administration, suggesting new avenues for the development of therapeutic strategies to ameliorate podocytopathy in DN.

### WIF1 deficiency in podocytes exacerbates renal injury in diabetic mice

**Fig. 5:**
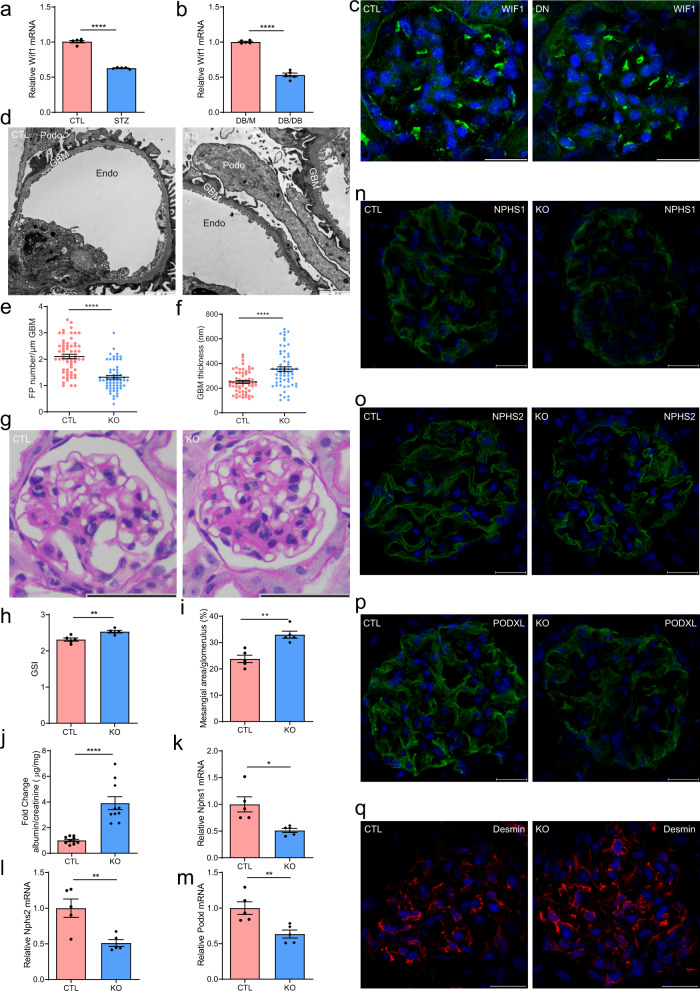
WIF1 deficiency in podocytes exacerbates renal injury in diabetic mice. **a** mRNA expression of Wif1 assessed by qRT–PCR in isolated glomerulus from control (CTL) and STZ-induced type I diabetic mice (STZ), 12 weeks after the last injection of STZ (*n* = 5 biologically independent animals, ****P < 0.0001). **b** mRNA expression of Wif1 assessed by qRT–PCR in isolated glomerulus from DB/M and DB/DB type 2 diabetic mice at 24 weeks of age (*n* = 5 biologically independent animals, ****P < 0.0001). **c** Immunostaining of WIF1 of kidney sections from control and DN (UNX/STZ) mice 12 weeks after the last injection of STZ. Scale bars, 20 μm. **d**–**f** TEM images of glomerular capillary loops of CTL (WT) and KO (Wif1 KO) mice (**d**) and quantification of FP number (**e**) and GBM thickness (**f**) 12 weeks after the last dose of STZ injection (*n* = 3 animals and 20 images per group, ****P < 0.0001). Scale bar, 2 μm. **g**–**i** PAS staining (**g**), GSI (**h**) and mesangial area expansion (**i**) in WT and Wif1 KO mice 12 weeks after the last dose of STZ injection (20 glomeruli per mouse were analyzed, *n* = 5 biologically independent animals per group, **P < 0.01). Scale bar, 50 μm. **j** Development of albuminuria (shown as fold change of the ratio of albumin to creatinine) after 12 weeks of the last injection of STZ (*n* = 10 biologically independent animals, ****P < 0.0001). **k**–**m** qRT-PCR analysis showing Nphs1 (**k**), Nphs2 (**l**), and Podxl (**m**) mRNA abundance in isolated glomerulus from diabetic WT and Wif1 KO mice (*n* = 5 biologically independent animals, *P < 0.05, **P < 0.01). **n**–**q** Immunofluorescence staining demonstrating the abundance and distribution pattern of NPHS1 (**n**), NPHS2 (**o**), PODXL (**p**), and desmin (**q**) in kidney sections of diabetic WT and Wif1 KO mice. Nuclei were visualized by DAPI. Scale bar, 20 μm. Data are presented as mean values ± SEM. Two-tailed unpaired Student’s *t* test was used for statistical comparisons (**a**, **b**, **e**, **f**, **h**–**m**). Source data are provided as a Source data file.

To investigate the possible role of WIF1 in the diabetic kidney, we assessed WIF1 expression in mouse models of STZ-induced type I diabetic mice and DB/DB type 2 diabetic mice. In comparison to control mouse glomerulus, WIF1 mRNA expression was significantly reduced in DN mice (Fig. 5a, b). WIF1 staining intensity was significantly higher in control mice, while dramatic loss of WIF1 staining was observed in DN mice (Fig. 5c). To assess the potential role of WIF1 in the development of DN, we induced diabetes by UNX combined with repeated STZ injections. Blood glucose levels, HbA1c levels, food intake, urine output, and body weight were not different between Wif1^ctrl^ and Wif1^podKO^ mice at baseline or 12 weeks after the induction of diabetes (Supplementary Table 2). Electron microscopy analysis revealed that Wif1^podKO^ mice experienced more severe podocyte injury, as evidenced by GBM thickening and podocyte FP broadening and effacement in the context of DN (Fig. 5d–f). Histological analysis showed greater mesangial expansion and a higher GSI in diabetic Wif1^podKO^ mice than in diabetic Wif1^ctrl^ mice (Fig. 5g–i). Consistent with the above observations, the level of albumin excretion by diabetic Wif1^podKO^ mice was significantly higher than that by Wif1^ctrl^ mice, suggesting that deletion of WIF1 exacerbated renal damage in diabetic mice (Fig. 5j). Significant downregulation of the expression of podocyte-associated molecules, including NPHS1 (Fig. 5k, n), NPHS2 (Fig. 5l, o), and PODXL (Fig. 5m, p) at both mRNA and protein levels was also observed in glomerulus of Wif1^podKO^ diabetic mice. Increased expression of desmin was also observed in diabetic Wif1^podKO^ mice compared with diabetic Wif1^ctrl^ mice (Fig. 5q). Together, these results suggest that the absence of WIF1 in podocytes aggravates proteinuria and podocyte damage in DN mice.

### Podocyte-specific loss of CLDN5 or WIF1 exacerbates interstitial fibrosis in a UUO mouse model

**Fig. 6:**
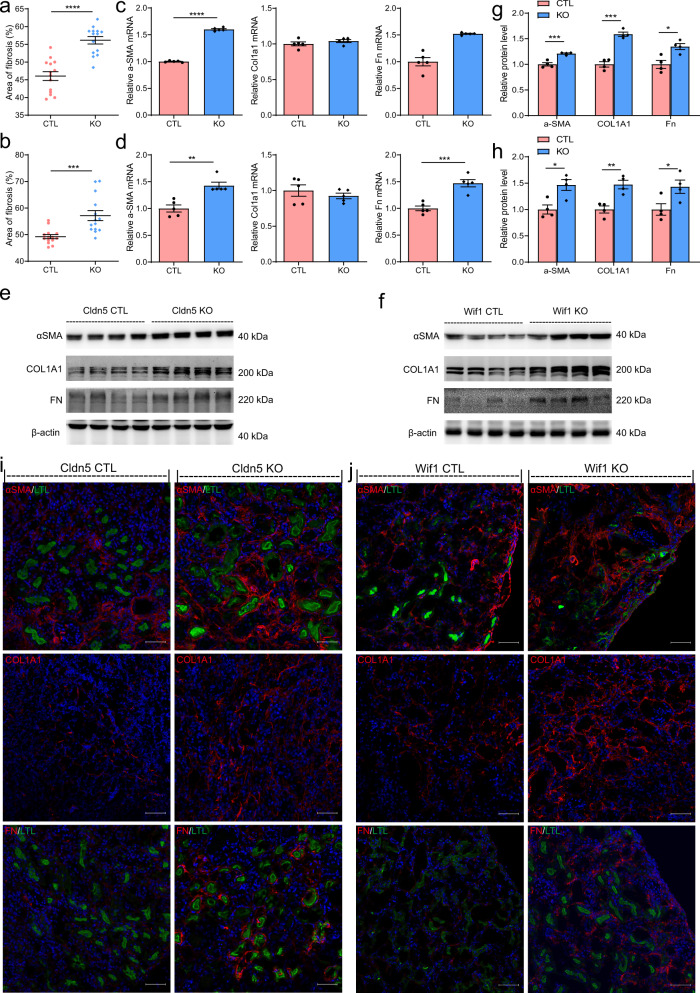
Podocyte-specific loss of CLDN5 or Wif1 exacerbates interstitial fibrosis 14 days following the UUO operation. **a**, **b** Relative area of fibrosis (%) in UUO-injured kidneys from the Cldn5 KO (**a**) and Wif1 KO (**b**) mice compared to their respective control mice measured using ImageJ (*N* = 7 biologically independent mice/group from two separate experiments, ***P < 0.001 and ****P < 0.0001). **c**, **d** qRT-PCR analysis of α-SMA, Col1a1 and Fn mRNA abundance in UUO-injured kidneys from the Cldn5 KO (**c**) and Wif1 KO (**d**) mice compared to their respective control mice (*n* = 5 biologically independent animals, **P < 0.01, ***P < 0.001, ****P < 0.0001). **e**, **f** Western blotting analysis of α-SMA, COL1A1, and FN in UUO-injured kidneys from Cldn5 KO (**e**) and Wif1 KO (**f**) mice compared to their respective control mice. **g**, **h** Bar graphs **g** for Cldn5 CTL and Cldn5 KO, **h** for Wif1 CTL and Wif1 KO) represent the mean ± SEM of fold changes corresponding to densitometric analyses. Densitometry calculations from four samples/group are shown and are normalized to β-actin as indicated (*P < 0.05, **P < 0.01, ***P < 0.001). **i**, **j** Immunofluorescence for α-SMA, COL1A1, and FN in kidney sections from the Cldn5 KO (**i**) and Wif1 KO (**j**) mice compared to those from their respective control mice. Scale bar, 50 μm. LTL (green) was used as a proximal tubule marker. Nuclei were visualized by DAPI. Data are presented as mean values ± SEM. Two-tailed unpaired Student’s *t* test was used for statistical comparisons (**a**–**d**, **g**, **h**). Source data are provided as a Source data file.

Our data indicated that the phenotypic changes in Cldn5^podKO^ podocytes aggravated interstitial fibrosis in a DN mouse model (Fig. 2k). Because WIF1 is predominantly expressed in podocytes, it could play a paracrine role on tubular epithelial cells through constant secretion into the preurine as a carrier. As WNT is an essential modulator of fibrosis development, we speculated that local production of WIF1 by podocytes might affect WNT pathway tone in proximal tubules and participate in the progression of fibrosis following acute kidney injury. In spite of the fact that genetic deletion is limited to podocytes, both Wif1^podKO^ and Cldn5^podKO^ kidneys showed increased fibrosis compared with their littermate controls 14 days after UUO induction according to various measures. The kidneys of knockout mice showed more severe renal interstitial fibrosis after UUO compared with that in controls, as shown by MTS (Supplementary Fig. 5a), Sirius Red staining (Supplementary Fig. 5b), and HE staining (Supplementary Fig. 5c). The size of interstitial fibrosis area was significantly increased in the knockout groups (Fig. 6a, b). Consistently, the mRNA levels of genes related to interstitial fibrosis and kidney injury (α-SMA, fibronectin (Fn), connective tissue growth factor (Ctgf), and Kim1) were markedly enhanced in knockout obstructed kidneys (Fig. 6c and d, Supplementary Fig. 5d, e). The protein levels of total α-SMA, COL1A1, FN, and KIM-1 were also increased, as determined by western blotting (Fig. 6e–h, Supplementary Fig. 5f, g) and immunofluorescence (Fig. 6I, j, Supplementary Fig. 5h, i). These results strongly suggest that disruption of WIF1 expression promotes UUO-induced renal fibrosis. Moreover, these effects were accompanied by upregulated WNT downstream genes expression including CCND1 and CD44. The levels of CCND1 and CD44 in the kidney were significantly increased in Cldn5^podKO^ mice with UUO, as determined by qRT-PCR, western blotting, and immunofluorescence (Supplementary Fig. 6a, c, e, g). Similar effects were also observed in Wif1^podKO^ mice (Supplementary Fig. 6b, d, f, h).

To directly demonstrate that podocytes secrete factors capable of silencing WNT signaling in proximal tubular cells, we assessed the levels of WNT target genes in cultured TKPTS cells exposed to podocyte culture medium (Supplementary Fig. 6i). We found that exposure of TKPTS to culture medium from Cldn5^podKO^ podocytes resulted in the increased expression of WNT target genes including Mmp7, Tcf7, Cd44, Lef1, and Ccnd1 in TKPTS (Supplementary Fig. 6j), compared with the culture medium from Cldn5^ctrl^ podocytes which had a higher concentration of WIF1 (Supplementary Fig. 6k). These data suggest that inadequate WIF1 secreted by podocytes in the Cldn5 and Wif1 knockout mice permits exaggerated kidney damage and fibrosis during UUO via WNT-dependent actions in tubular epithelial cells.

### Systemic delivery of WIF1 attenuates DN and UUO-induced renal fibrosis

**Fig. 7:**
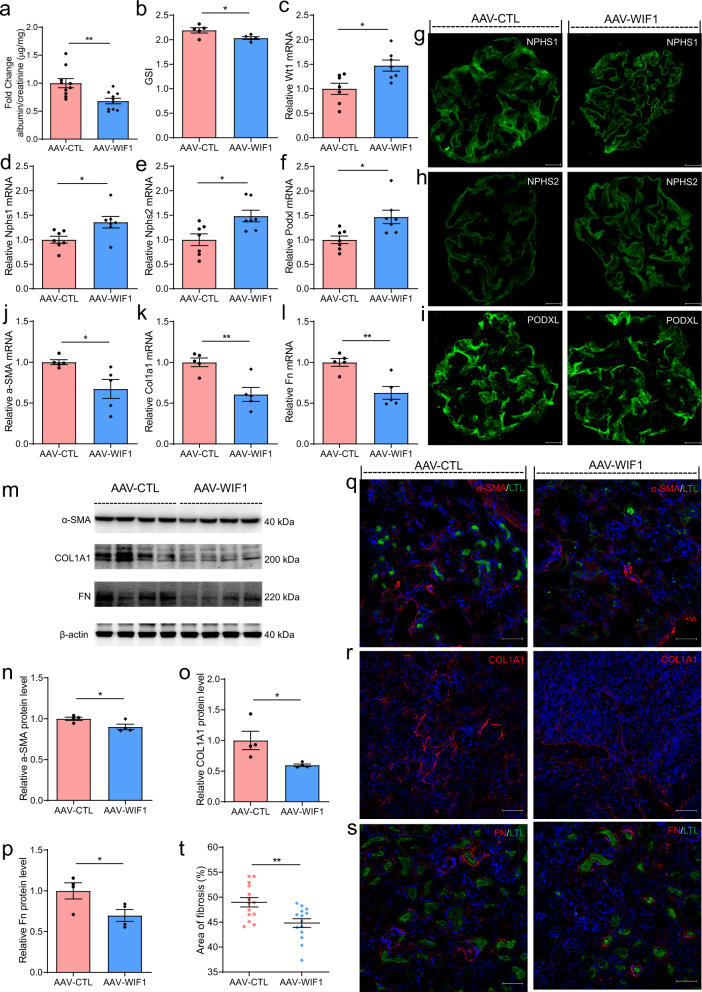
Systemic administration of WIF1 exacerbates glomerular injury in diabetic mice and fibrosis in UUO mice. **a** Development of albuminuria (shown as the fold change in the ratio of albumin to creatinine) in AAV-CTL and AAV-WIF1 treated mice after 12 weeks of the last injection of STZ (*n* = 10 biologically independent animals, **P < 0.01). **b** GSI in kidneys from the AAV-CTL and AAV-WIF1 treated mice 12 weeks after the last dose of STZ injection (20 glomeruli per mouse were analyzed, *n* = 5 biologically independent animals per group, *P < 0.05). **c**–**f** Glomerular gene expression of Wt1 (**c**), Nphs1 (**d**), Nphs2 (**e**), and Podxl (**f**) (*n *= 7 biologically independent animals, *P < 0.05). **g**–**i** Representative immunofluorescence images for NPHS1 (**g**), NPHS2 (**h**), and PODXL (**i**) in kidney sections from the AAV-CTL and AAV-WIF1 treated mice. Scale bar, 20 μm. **j**–**l** Kidney gene expression of α-SMA, Col1a1 and Fn (*n* = 5 biologically independent animals, *P < 0.05, **P < 0.01). **m**–**p** Western blotting analysis of α-SMA, COL1A1, and FN in kidneys from the AAV-CTL and AAV-WIF1 treated mice. Densitometry calculations are normalized to β-actin as indicated (*n* = 4 biologically independent animals, *P < 0.05). **q**–**s** Immunofluorescence for α-SMA, COL1A1, and FN in kidney sections from the AAV-CTL and AAV-WIF1 treated mice. Scale bar, 50 μm. **t** Relative area of fibrosis (%) measured using ImageJ (**P < 0.01). *n* = 7 biologically independent mice/group from two separate experiments. Data are mean ± SEM. Data are presented as mean values ± SEM. Two-tailed unpaired Student’s *t* test was used for statistical comparisons (**a**–**f**, **j**–**l**, **n**–**p**, **t**). Source data are provided as a Source data file.

Does exogenous WIF1 hold promise as a therapeutic agent for injured kidney? We tested the therapeutic potential of systemic delivery of WIF1 in type 1 diabetic mice. Treatment of diabetic mice with AAV-WIF1 significantly attenuated albuminuria by 12 weeks (Fig. 7a). Although there was some variation from mouse to mouse in these functional studies, the results of three independent experiments suggested that systemic delivery of WIF1 alleviated albuminuria in diabetic mice. The degree of glomerulosclerosis was also less severe in the WIF1 treatment group compared with control group (Fig. 7b).qRT-PCR and immunofluorescence showed that the levels of key podocyte molecules, including WT1 (Fig. 7c), NPHS1 (Fig. 7d, g), NPHS2 (Fig. 7e, h), and PODXL (Fig. 7f, i), were significantly increased in diabetic mice injected with AAV-WIF1 compared with those injected with AAV-CTL. Taken together, these results demonstrate that WIF1 can significantly attenuate the increases in parameters of glomerular injury in diabetic mice in vivo.

Next, we tested whether circulating WIF1 can also protect mice against the development of fibrosis in UUO model mice. WIF1 was delivered after disease onset (4 days after UUO induction), and fibrosis extent was assessed 14 days after UUO induction. qRT-PCR showed that the mRNA levels of α-SMA (Fig. 7j), Col1a1 (Fig. 7k), and Fn (Fig. 7l) were significantly decreased in UUO mice injected with AAV-WIF1 compared with those injected with AAV-CTL. Similar to the results obtained by western blotting, which indicated that the protein levels of α-SMA (Fig. 7m, n), COL1A1 (Fig. 7m, o), and FN (Fig. 7m, p) were reduced, immunofluorescence staining revealed that the sizes of the areas positive for α-SMA (Fig. 7q), COL1A1 (Fig. 7r), and FN (Fig. 7s) were decreased in AAV-WIF1 treated mice compared to AAV-CTL treated mice. The fibrosis score was also significantly reduced upon WIF1 application (Fig. 7t). Collectively, these findings indicate that WIF1 treatment can markedly ameliorate tubulointerstitial fibrosis in UUO model mice.

### CLDN5 interacts with ZO1 and ZONAB in podocytes

**Fig. 8:**
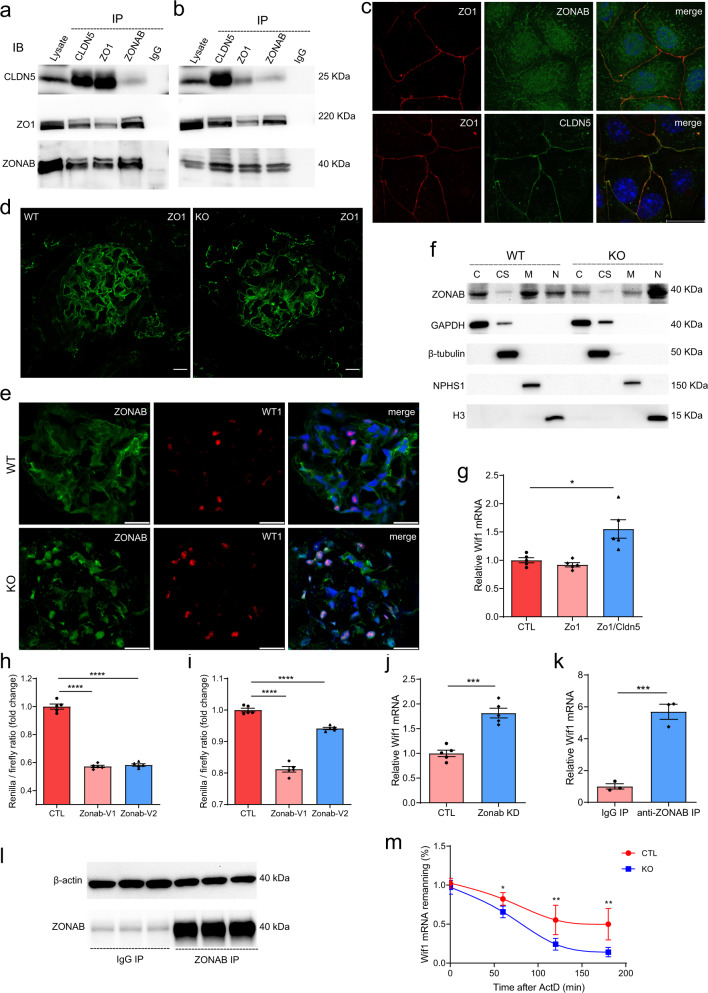
Regulation of Wif1 expression by CLDN5. **a** Co-IP of CLDN5, ZO1, and ZONAB in multiply transfected HEK293 to determine CLDN5 and ZO1/ZONAB interaction. **b** Co-IP showing that endogenous CLDN5 interacts with endogenous ZO1/ZONAB in isolated mouse glomerulus. Antibodies used for immunoprecipitation are shown above the lanes; antibody for blot visualization is shown on the left. **c** Double immunostaining for ZO1/ZONAB and ZO1/CLDN5 in primary podocytes plated on coverslips. **d** ZO1 immunostaining in kidney sections from WT and Cldn5 KO mice. **e** Double immunostaining for ZONAB and WT1 in kidney sections from WT and Cldn5 KO mice. **f** Western blotting for ZONAB expression in the glomerulus of WT and Cldn5 KO mice. Glomerulus were isolated from WT and Cldn5 KO mice followed by fractionation. The levels of ZONAB were analyzed by western blotting, with GAPDH, β-tubulin, Nephrin, and Histone H3 serving as controls for the purity of the cytosolic fraction (C), cytoskeletal fraction (CS), membrane fraction (M), and nuclear fraction (N), respectively. **g** Expression levels for Wif1 mRNA in Cldn5 KO primary podocytes transfected with empty vector pcDNA3 (CTL), expression vector for Zo1, or co-transfected with expression vector for Zo1 and Cldn5 (*n* = 5 independent experiments, *P < 0.05). **h**, **i** Renilla/firefly luciferase activity ratios in MDCK (**h**) and primary mouse podocytes (**i**) transfected with Wif1:3′-UTR and empty vector pCMV6 (CTL), pCMV6-Zonab-V1, or pCMV6-Zonab-V2 (*n* = 5 independent experiments, ****P < 0.0001). **j** Effects of transfection with an siRNA targeting Zonab (Zonab KD) on Wif1 gene expression in primary podocytes from Cldn5 KO mice (*n* = 5 independent experiments, ***P < 0.001). **k**, **l** Association of endogenous ZONAB with endogenous Wif1 mRNA as measured by RIP and qRT-PCR analysis using either an anti-ZONAB antibody or control IgG (**k**). The values are the means ± SEM from triplicate samples, ***P < 0.001 compared with IgG IP. ZONAB protein levels were analyzed by western blotting to ensure efficient pull-down (**l**). **m** Levels of the Wif1 mRNA at different times after administration of actinomycin D (*n* = 5 independent experiments, *P < 0.05, **P < 0.01). Nuclei were visualized by DAPI. Scale bar, 20 μm. Data are presented as mean values ± SEM. Two-tailed unpaired Student’s *t* test was used for statistical comparisons (**g**–**k** and **m**). Source data are provided as a Source data file.

On the basis of the information currently available, ZO1 forms scaffolds to anchor TJ membrane proteins, and it also plays very important role in controlling gene expression via binding to and tuning the activity of the transcription factor ZONAB^14,15^. To directly evaluate the interaction between CLDN5 and the ZO1/ZONAB complex, we performed co-immunoprecipitation (CoIP) in sparsely plated HEK293 cells transfected with four genes simultaneously. In HEK293 cells multiply transfected with CLDN5, ZO1, ZONAB variant 1, and ZONAB variant 2, an anti-CLDN5 antibody precipitated ZO1 and ZONAB; reciprocally, an anti-ZO1 antibody coimmunoprecipitated CLDN5 and ZONAB, and a ZONAB antibody coimmunoprecipitated ZO1 and CLDN5 (Fig. 8a). To test whether CLDN5 and ZO1/ZONAB are associated with native tissue, glomerular extracts were immunoprecipitated with anti-ZO1, anti-CLDN5, and anti-ZONAB antibodies, and immunoprecipitation of the three proteins was monitored by immunoblotting. We confirmed the endogenous interaction between CLDN5, ZO1, and ZONAB in glomerular extracts (Fig. 8b). Protein–protein interactions are also reflected by co-localization of two or more proteins in the same subcellular location when resolved at sufficient resolution. Under fluorescence microscopy, we observed that CLDN5, ZO1, and ZONAB were localized to plasma membrane in cultured WT primary podocytes (Fig. 8c). In addition to its membrane localization, ZONAB was also constitutively found in the cytoplasm and nucleus (Fig. 8c). Together, these data reflect the existence of a complex containing CLDN5, ZO1, and ZONAB in podocytes.

### Podocyte-specific CLDN5 deficiency increases ZONAB nuclear localization

We next asked whether Cldn5 knockout deregulates ZO1/ZONAB signaling in podocytes. A marked reduction and more fragmented of ZO1 expression were observed in the Cldn5 KO mice compared with their wild-type littermates (Fig. 8d). qRT-PCR showed that the mRNA level of ZO1 was not significantly changed after Cldn5 deletion (Supplementary Fig. 7a), suggesting that the downregulation of ZO1 expression occurred at the protein level, possibly due to higher levels of degradation. Confocal imaging showed membrane, cytoplasmic and weak nuclear ZONAB labeling in Cldn5^ctrl^ mouse glomerulus and distinct colocalization of ZONAB with WT1 in Cldn5^podKO^ mice (Fig. 8e), which indicated that CLDN5 deletion in podocytes increased ZONAB nuclear localization. Subcellular fractionation of the isolated glomerulus, followed by western blotting analysis, indicated that CLDN5 deletion and ZO1 reduction in podocytes promoted ZONAB to undergo nuclear translocation (Fig. 8f). We then determined whether the effects of CLDN5 deletion on Wif1 expression could be rescued by re-introduction of ZO1. Co-transfection of CLDN5-deficient primary podocytes with Zo1 and Cldn5, but not transfection with Zo1 alone, increased Wif1 expression (Fig. 8g), indicating that CLDN5 and ZO1 functionally interact to regulate Wif1. Taken together, our data so far indicate that CLDN5 forms a complex with ZO1 and ZONAB in normal podocytes under physiological conditions, and this complex is required to sustain ZONAB’s subcellular localization and adequate levels of WIF1 to maintain normal WNT signaling activity.

### ZONAB regulates Wif1 expression through the 3′-UTR of Wif1

We next analyzed the molecular mechanism by which ZONAB reduces the expression of Wif1 mRNA. To evaluate whether ZONAB plays a transcriptional role on Wif1 promoter, we performed reporter gene assays by transiently transfecting primary podocytes and MDCK cells with a plasmid construct containing the firefly luciferase gene driven by the Wif1 promoter fragments together with Zonab or its vector control. This analysis showed little to no effect on Wif1 mRNA (Supplementary Fig. 7b, c), suggesting that the regulatory effect of ZONAB on Wif1 mRNA expression is not mediated by the 5′-promoter of Wif1. Next, to understand whether ZONAB targets the Wif1 3′-UTR, we generated the reporter constructs containing the entire mouse Wif1 3′-UTR sequence cloned downstream of the Renilla luciferase gene. The reporter was transfected with Zonab into primary podocytes and MDCK cells. Luciferase activities were decreased in both cell types following co-transfection with a ZONAB expression vector (Fig. 8h, i), indicating that ZONAB acts as a repressor of Wif1 expression via targeting the 3′-UTR of Wif1. To verify that ZONAB is capable of inhibiting endogenous WIF1 in native podocytes, we knocked down Zonab with a specific siRNA and analyzed the expression level of Wif1 via qRT-PCR. Following knockdown of Zonab mRNA, qRT-PCR revealed significantly elevated levels of Wif1 transcript in Cldn5^podKO^ podocytes compared to cells treated with control siRNA (Fig. 8j). Taken as a whole, our results establish that transcriptional regulation of WIF1 by ZONAB at least in part is brought about via repression of the Wif1 3′-UTR.

Next, we examined whether ZONAB directly interacted with Wif1 mRNA in glomerulus. RIP analysis using an anti-ZONAB antibody followed by measurement of Wif1 mRNA levels by qRT-PCR analysis in the immunoprecipitate material revealed that Wif1 mRNA associated with ZONAB, as Wif1 mRNA was highly enriched in the ZONAB immunoprecipitate relative to the IgG immunoprecipitate (Fig. 8k, l). Next, we examined the effects of ZONAB on the stability of endogenous Wif1 mRNA. Here, we treated WT and Cldn5 KO primary podocytes with the transcriptional inhibitor actinomycin D and measured the half-lives of Wif1 transcripts using qRT-PCR analysis. We found that Cldn5 deficiency in podocytes, which increased the nuclear localization of ZONAB, decreased Wif1 mRNA levels by enhancing its degradation, since Cldn5 KO caused a faster decline in Wif1 mRNA levels (Fig. 8m), this was indicated by the fact that Wif1 mRNA was less stable when CLDN5 levels were diminished. Thus, we conclude that ZONAB can bind to and destabilize endogenous Wif1 mRNA transcripts in podocytes. ZONAB preferentially binds an RNA Y-box protein recognition sequence (YRS) ([UAC][CA]CA[UC]C[ACU]) in vitro^16^. Sequence analysis revealed that mouse Wif1 transcript has two such potential binding sites in its 3′-UTR (Supplementary Fig. 7d). However, deletion of either one or both of the predicted binding sites in Wif1 3′-UTR could not abolish the effect of ZONAB (Supplementary Fig. 7e).

## Discussion

First, we showed that mice with podocyte-specific deletion of Cldn5 manifested albuminuria but not glomerulosclerosis, as least within our observation period. When we looked at kidney under the electron microscope, TEM revealed the presence of thickening of the GBM and mild FP effacement. Furthermore, we observed a significant reduction in CLDN5 expression in two DN mouse models. It has been shown that the TJs scaffold protein ZO1 is likewise reduced under the same conditions^17^, further study is required to determine whether reduced expression of ZO1 is a consequence of diminished podocyte CLDN5 in these diseases, or vice versa. Crucially, in two DN models, animals with a podocyte-specific deletion of CLDN5 exhibited a more severe nephropathy, including more proteinuria, and evidence of more marked podocyte injury, but interestingly also more severe interstitial fibrosis. These results indicate that CLDN5 is a critical injury response gene in podocytes with a strong linkage to kidney injury.

In an attempt to determine the underlying mechanism, we found that Cldn5 deletion in podocytes repressed expression of Wif1. WIF1 is a secreted protein that functions as a paracrine inhibitor of the WNT signaling pathway by binding, and inhibiting the activity of extracellular WNT ligands^18^. Aberrant activation of WNT/β-catenin signaling plays a central role in the pathogenesis of a wide variety of kidney diseases^19,20^. The results of the present study show that the deletion of CLDN5 leads to activated WNT/β-catenin signaling in podocytes via downregulation of Wif1 expression, resulting in a similar base-line phenotype to the podocyte-specific stabilized Ctnnb1 expression mice, a mouse model with increased expression of Ctnnb1 via being rendered into a stabilized and dominant active form by deleting the phosphorylation site^21^. More importantly, targeted delivery of Wif1 obviated the development of DN in Cldn5 knockout mice. Ultrastructural examination of podocyte-specific conditional Wif1-knockout kidneys showed irregular thickening of the GBM with occasional regions of FP effacement, mimicking the phenotypes of Cldn5-deficient podocytes. Therefore, our results establish that CLDN5 is a previously undescribed regulator of WNT/β-catenin signaling activity in podocytes.

We demonstrate that WIF1 is expressed specifically in glomerular podocytes, and that its expression is decreased in diabetic glomerular disease models. Strikingly, our observations also uncover an important role for Wif1 deficiency in promoting podocyte injury and glomerulosclerosis during DN, and show that systemic administration of Wif1 slightly reduced podocyte injury in a DN model. A recent study identified a loss-of-function mutation in Wif1 as a potential novel cause of a Nail-Patella-like disorder^22^. Nail-patella syndrome (NPS) is a rare autosomal dominant disorder that is primarily characterized by dysplastic nails and missing or hypoplastic patellae^23^. One-third to one-half of the patients also suffer from renal issues^24^. The main pathology involves ultrastructural abnormalities in the GBM^24^, that are similar to the ultrastructural changes seen in the kidney of podocyte-specific Wif1 KO mice. Therefore, studies using Wif1 mutant mice provide valuable information for better understanding the glomerular phenotypes seen in kidneys of NPS patients.

In addition, we identified WIF1 as a factor secreted by podocytes that may also contribute to tubular dysfunction in chronic kidney disease. The progression of primary glomerular disease to induce tubulointerstitial lesions is well established, the mechanisms by which glomerular injury extends to proximal tubules are still under investigation. Here we found that blockade of WIF1 secretion by genetic deletion of Cldn5 or Wif1 in podocytes markedly enhanced renal fibrosis after UUO. This effect was associated with increased WNT downstream gene expression. Systemic delivery of WIF1 using an adenoviral delivery system profoundly inhibited the development and progression of kidney fibrosis using the UUO model. In vitro, exposure of TKPTS to culture medium from Cldn5-deficient podocytes resulted in the increased expression of WNT target genes, compared with the culture medium from wild-type podocytes which has higher concentration of WIF1. Collectively, these data add strong support for a potential mechanism to account for how glomerular injury triggers tubular damage during chronic kidney disease progression.

Some of the preliminary evidence that ZO proteins participated in the control of gene expression was the observation that the transcription factor ZONAB was expressed in the nucleus and also co-localized with ZO1 to form a complex^14,25,26^. We show here that the interaction of ZO1 with CLDN5 may be important for the stabilization of ZO1 in podocytes via anchoring of ZO1 to the plasma membrane to inhibit the nuclear accumulation of ZONAB. ZONAB localization in podocytes is affected by Cldn5 knockout along with biochemical interactions between CLDN5, ZO1, and ZONAB. And altered ZO1 distribution in Cldn5 knockout mice provides increasing evidence for functional interactions between these proteins. Loss of CLDN5 likely disrupts this membrane protein complex and induces the translocation of ZONAB into the nucleus, leading to downregulation of WIF1 expression. Even though ZO1 has been identified as an important component of the SD complex and podocyte-specific deletion of ZO1 gene impaired SDs formation^27^, we did not observe bona fide SD morphological defect in our animals despite a significant reduction in ZO1 expression. We do not currently know why such a difference exists, one possibility is that the signal strengths generated by the CLDN5 deficiency are different from ZO1 knockout, leading to their different outcome.

ZONAB has been reported to be involved in transcriptional activation and repression, as well as in posttranscriptional mechanisms of gene expression regulation, including mRNA packaging, transport, localization and stability^14,25,28^. Here, we show that ZONAB influences Wif1 expression predominantly via the Wif1 3′-UTR in podocytes, but does not affect the promoter activity of Wif1. The Wif1 mRNA is a target of ZONAB and the association between ZONAB and Wif1 mRNA decreases Wif1 mRNA stability. Although ZONAB has been proved to act as a positive regulator of mRNA abundance by binding to 3′-UTRs, we show here that the transcriptional regulation of WIF1 by ZONAB occurs via 3′-UTR-mediated repression. Our observations are consistent with a recent study demonstrating both reductions and increases in transcripts levels in cells with ZONAB depletion via integrated proteomic and transcriptomic analysis^29^. Although ZONAB, as a nucleic acid-binding protein that directly regulates gene expression, has been shown to preferentially bind to YRS in RNA, the binding sites deletion caused no difference in Wif1 mRNA levels. One possibility is that the regulation of Wif1 by ZONAB occurs through a non-sequence-specific mechanism.

In conclusion, the present study reveals a previously undescribed function and an important regulatory role for the TJ protein CLDN5 in restricting WNT activity in the kidney (Supplementary Fig. 8). The paradigm of CLDN5 restricting WNT signaling can therefore be extended to other sites of CLDN5 expression. Interestingly, CLDN5 is regulated by WNT signaling^30^, suggesting that it might participate in a negative feedback loop to limit WNT-initiated signals. Our experiments also provide proof of principle that CLDN5 and WIF1 might be developed into therapeutic modalities for the treatment of kidney diseases affecting millions of people worldwide.

## Methods

### Antibodies, plasmids, and cell lines

The antibodies used in this study are summarized in Supplementary Table 3 and Supplementary Table 4. The full-length cDNA of mouse Zo1 (NM_009386) was synthesized and cloned into pLVX vector by Sango Biotechnology (China). The expression plasmids containing mouse Cldn5 (pCMV6-Cldn5, NM_013805), Zonab transcript variant 1 (pCMV6-Zonab-V1, NM_139117), and Zonab transcript variant 2 (pCMV6-Zonab-V2, NM_011733) genes were purchased from Origene Technologies (USA). MDCK II, human HEK293 cells, 3T3-L1, and mouse proximal tubule cells (TKPTS) were obtained from ATCC (USA) and cultured according to the distributor’s recommendations.

### Animals

Mice were housed in a standard environment which was characterized by 12 h light/dark cycle, 22–25 °C, and 40–60% humidity with free access to water and chow. All animals were maintained in specific pathogen-free facilities. The 12-week-old male DB/M and DB/DB were obtained from Cavens Biogle (China). C57BL/6J (B6) mice were obtained from Vital River Laboratory (China).

For the generation of Cldn5^loxP/loxP^ mice, targeting vector was constructed by inserting one SDA (self-deletion anchor)-flanked neomycin cassette and two loxP sites flanking the first exon of Cldn5 and then electroporating into embryonic stem cells from C57BL/6J mice (conducted by Cyagen Biosciences Inc, China). With one subsequent cross with B6 animals, the Neo transgene was removed and the obtained Cldn5^loxP/+^ mice were then intercrossed in order to generate the Cldn5^loxP/loxP^ mice. For Cldn5^loxP^ genotyping, a PCR with the following primers was performed F2: 5′-CCTCTGGCTAGTGTGGCTCTAAAC-3′, R2: 5′-GCATTCCTGACTCTGGCCTTTTAG-3′, resulting in a 328 bp band in the case of Cldn5^loxP^ mice, and 215 bp for wild-type. Same strategy was used to generate the Wif1^loxP/loxP^ mice with exon 3 selected as conditional knockout region. The primer sequences for genotyping the Wif1^loxP^ allele were F2: 5′-CATTGATGACCCAAGTTGCTGAG-3′ and R2: 5′-TTGAACCATGCTTGGAGAGAAGAG-3′, which yielded a 205-bp band for the WT allele and a 253-bp band for the Wif1^loxP^ allele.

Cldn5^loxP/loxP^ mice or Wif1^loxP/loxP^ mice were crossed with Nphs2-Cre mice (Jackson Laboratory, 008205) to generate podocyte-specific Cldn5 knockout mice Nphs2-Cre^+/−^/Cldn5^loxP/loxP^ (Cldn5^podKO^) or podocyte-specific WIf1-knockout mice Nphs2-Cre^+/−^/Wif1^loxP/loxP^ (Wif1^podKO^), respectively. Heterozygous with Nphs2-Cre positive littermates served as controls. Nphs2-Cre transgene was detected by amplifying a 200 bp region of Cre recombinase by PCR. Forward and reverse oligonucleotides used were, respectively, 5′-CGGTTATTCAACTTGCACCA--3′ and 5′-GCGCTGCTGCTCCAG-3′.

R26-stop-EYFP reporter mice harbor a conditional Enhanced Yellow Fluorescent Protein (EYFP) allele that requires Cre mediated recombination for expression. To facilitate the isolation of primary podocytes by fluorescence-activated cell sorting (FACS), we crossed R26-stop-EYFP mutant mice (Jackson Laboratory, 006148) with Cldn5^podKO^ mice to get podocyte-specific Cldn5 knockout reporter mice (Nphs2-Cre^+/−^/Cldn5^loxP/loxP^/EYFP-stop^loxP/loxP^) and control reporter mice (Nphs2-Cre^+/−^/Cldn5^loxP/-^/EYFP-stop^loxP/loxP^). The forward and reverse oligonucleotides for genotyping R26-stop-EYFP mutant mice were, respectively, 5′-AGGGCGAGGAGCTGTTCA-3′ and 5′-TGAAGTCGATGCCCTTCAG-3′, which generated a 384 bp band.

### STZ-induced DN

UNX combined with STZ were used to accelerate the development of DN. Briefly, after a 1-week recovery period from UNX, STZ was injected into 5–7-week-old fasting wild-type C57BL/6, Cldn5^ctrl^, Cldn5^podKO^, Wif1^ctrl^, and Wif1^podKO^ mice (intraperitoneal injection of 50 mg/kg body weight) for 5 days.

Spot samples of urine were collected every 4 weeks. Urinary albumin excretion rates were analyzed 4, 8, and 12 weeks after the last injection of STZ. Kidneys were harvested and processed for histological and ultrastructural analyses, glomerulus were collected for qRT-PCR and western blotting analysis after the 12-week follow-up.

### In vivo administration of recombinant AAV

rAAV9 is the most efficient rAAV serotype for kidney gene delivery^31^. To overcome the non-specificity of rAAV9, we utilized the rAAV9 vector containing the Nphs1 promoter to drive expression of WIF1 in podocytes. Four to six weeks Cldn5^podKO^ mice or wild-type CTL mice were performed with UNX and injected with STZ as described above. After 4 weeks of STZ injection, mice received in situ renal injection with 1 × 10^12^ genomic particles of rAAV9-NPHS1-GFP (AAV-CTL) or rAAV9-NPHS1-WIF1 (AAV-Wif1) (prepared by Hanbio Biotechnology Inc, China) at five independent points. For systemic Wif1 gene delivery, rAAV9-CMV-GFP (AAV-CTL) or rAAV9-CMV-WIF1 (AAV-Wif1) (prepared by Hanbio Biotechnology Inc, China) was administered to the diabetic mice 4 weeks after STZ injection via tail vein injection. Spot samples of urine were collected every 4 weeks. All mice were sacrificed to harvest kidneys 8 weeks after transduction.

### UUO

Eight- to ten-week-old male mice were anesthetized and the left kidney exposed by the retroperitoneal approach. The ureter was ligated with 4-0 silk suture at 2 points, close to the renal pelvis. Kidneys were harvested for analysis 14 days after UUO. In the adenovirus administration experiments, 1 × 10^12^ genomic particles of either rAAV9-CMV-GFP (AAV-CTL) or rAAV9-CMV-WIF1 (AAV-Wif1) was given by tail vein on day 4, relative to the date of surgery.

### Urine and serum analyses

Urinary albumin and creatinine were measured using mouse albumin-specific ELISA (Bethyl Laboratories, USA) and Quantichrome Creatinine Assay Kit (Nanjing Jiancheng, China). Proteinuria was expressed as μg albumin/mg creatinine. Blood glucose was measured using Yuwell Blood Glucose Meter (China). Plasma cystatin C was analyzed by Cystatin C Mouse ELISA Kit (Sango Biotechnology, China). Plasma HbA1c was measured by Hemoglobin A1c (HbA1c) (Mouse) ELISA Kit (BioVision, USA).

### Histologic analysis

Paraffin-embedded mouse kidney sections (5-μm thickness) were prepared by a routine procedure. Sections were stained with Hematoxylin and Eosin (H&E), Sirius Red, PAS, and MTS. Histology images were collected by cellSens Software v3.1. PAS-stained sections were examined for the degree of glomerular damage (GSI and mesangial area expansion) using a semiquantitative scoring method as described by Maric et al.^32^. Image J v1.8.0 was used to quantify PAS staining micrographs. MTS sections were examined for the degree of tubulointerstitial fibrosis using a semiquantitative scoring method as described by Maric et al.^32^. TEM was performed on glutaraldehyde-fixed, epoxy-embedded kidney samples and stained with uranyl acetate and lead citrate. TEM images were collected by RADIUS Software v2.1. To determine the GBM thickness and the number of FP per µm of GBM, TEM images were analyzed using NIH ImageJ v1.8.0 software.

### Immunofluorescence staining

For immunofluorescence of kidney tissue, 8-μm frozen sections were fixed in ice-cold methanol or acetone. For immunofluorescence of cultured cells, primary podocytes on collagen I coated coverslips were fixed with 4% paraformaldehyde. Then incubated with the appropriate primary antibodies after blocking with 10% FBS in PBS, developed using FITC and/or rhodamine (TRITC) conjugated secondary antibodies (Millipore), and mounted with ProLong™ Gold Antifade Mountant. Immunofluorescence images were collected and processed by Zen Software v2.3 (blue edition).

### RNA-seq

Total RNA was extracted using Trizol reagent (Invitrogen, 15596018) from glomerulus of Cldn5^ctrl^ and Cldn5^podKO^ mice at 4 weeks old. RNA-seq was performed on an Illumina Novaseq platform by Annoroad Genome (China). Paired-end clean reads were aligned to the mouse reference genome (Ensemble_GRCm38.90) with TopHat (version 2.0.12), and the aligned reads were used to quantify mRNA expression by using HTSeq-count (version 0.6.1). Differential expression analysis of two groups was performed using the DESeq2 R package (1.16.1). The resulting *P*-values were adjusted using the Benjamini and Hochberg’s approach for controlling the false discovery rate. Genes with an adjusted *P*-value <0.05 and a fold-change >2.0 were assigned as differentially expressed.

### Primary podocytes isolation and treatment

Glomerulus were isolated from the Cldn5^ctrl^ and Cldn5^podKO^ mice expressing a GFP-reporter, by using Dynabeads M-450 Tosylactivated (#14013, Invitrogen) perfusion. The glomerulus was then further digested enzymatically (Multi Tissue Dissociation Kits, Miltenyi) and dissociated into a single-cell suspension by using the gentleMACS™ Dissociators, and FACS was used to isolate the GFP positive podocytes. For podocytes culture, RPMI 1640 supplemented with 10% fetal calf serum and 3T3-L1 medium were mixed in a 1:1 ratio as described^33^. The expression plasmid containing mouse Zo1 cDNA (pLVX-Zo1) was transfected alone or together with Cldn5 (pCMV6-Cldn5) into the Cldn5-deficient podocytes using lipofectamine 2000 (Invitrogen). For Zonab siRNA treatment, a pool of three target-specific 21 nt siRNA duplexes (#sc-38632, Sango Biotechnology, China) were designed against the coding region of mouse Zonab gene (NM_139117, NM_011733). A scrambled siRNA duplex (#sc-37007, Sango Biotechnology, China) was used as negative control. Either Zonab siRNA or scrambled siRNA was transfected to primary podocytes in 12-well culture dishes with lipofectamine 2000 for 24 h. For immunofluorescence experiment, isolated glomerulus were cultured on type I collagen-coated culture dishes for three days. After removing the remaining glomerular cores, podocytes cultured on glass coverslips were fixed in methanol, and processed for double-label immunofluorescence microscopy using appropriate antibodies. For actinomycin D (Sigma, USA) treatments, podocytes were treated with 5 μg/ml of actinomycin D or with the appropriate amount of carrier (dimethyl sulfoxide).

### TKPTS cells experiment

In conditioned medium experiments, FACS-sorted WT or Cldn5 KO primary podocytes were cultured for 24 h. Supernatants were collected and cell debris was removed by centrifugation (2000 × *g*, 5 min). TKPTS cells were stimulated with 50% podocyte supernatants, added to their usual culture medium for 12 h (Supplementary Fig. 6i). RNA was subsequently extracted, and real-time qPCR was performed.

### RNA extraction and qRT-PCR

Total RNA was extracted from isolated glomerulus or cultured cells using Trizol (Ambion, USA). RNA was reverse transcribed by using a reverse transcription system kit according to the instructions of the manufacturer (Takara, Japan) followed by qRT-PCR amplification using SYBR Green PCR Master Mix (ABI, USA) and the Thermo Fisher QuantStudio3 system. qRT-PCR results were acquired by QuantStudioTM Design & Analysis SE Software v1.5.0. Supplementary Table 5 contains the primer sequences used in this study. The expression levels of each mRNA were calculated after normalizing to those of *β*-actin. Results were expressed as 2^−ΔCt^ values with ΔCT = Ct_gene_ − Ct_*β*-actin_.

### Western blotting analysis

Total protein extracts were obtained by lysing isolated glomerulus or whole kidney in 1× Laemmli buffer (50 mM Tris-Cl pH 7.5, 2% SDS, 10% glycerol, 5% β-mercaptoethanol, 0.01% bromophenol blue). Cellular fractions (membrane, cytoskeleton, cytosolic, and nuclear) obtained from glomerulus were extracted using the Chemicon Compartmental Protein Extraction Kit according to the manufacturer’s instructions (Millipore). Protein expression was detected by SDS/PAGE under reducing conditions, and immunoblotting was performed with antibodies. HRP-conjugated secondary antibodies (Thermo Fisher) followed by ECL (Thermo Fisher) incubation allowed protein band detection. The integration of all blots images was performed on Adobe Illustrator 2021. Image J v1.8.0 was used to quantify western blotting results.

### Luciferase reporter assay

The mouse Wif1 gene promoters (2 kb, 1 kb, and 0.5 kb before start codon of the Wif1 open reading frame, synthesized by Shanghai Sango Biotechnology) were cloned into pGL3-Basic luciferase reporter vector (Promega) with NheI and HindIII sites. The pGL3 reporter, the pGL4.74 Renilla luciferase control vector (Promega), and pCMV6-Zonab vector were co-transfected to MDCK cells and primary podocytes in 96-well culture dishes using lipofectamine-2000. The 771 bp 3′-UTR of mouse Wif1 gene (synthesized by Shanghai Sango Biotechnology) was inserted into the psiCHECK-2 (Clontech) downstream of the luciferase gene using XhoI/NotI. The psiCHECK-2-Wif1: 3′-UTR and pCMV6-Zonab vector were co-transfected to MDCK cells and primary podocytes in 96-well culture dishes using lipofectamine 2000. The mutant 3′-UTR (deletion of predicted ZONAB binding site CCCCACCA, or CCCATCT, or both) of mouse Wif1 gene (synthesized by Shanghai Sango Biotechnology) were inserted into the psiCHECK-2 downstream of the luciferase gene using XhoI/NotI. Wild-type or mutant psiCHECK-2-Wif1: 3′-UTR and Zonab siRNA were co-transfected to primary podocytes in 96-well culture dishes using Lipofectamine 2000. Twenty-four hours after transfection, firefly and renilla luciferase activities were measured with a chemiluminescence reporter assay system—Dual Glo (Promega) in Fluostar Omega (BMG). Luciferase activities were collected by Omega Software v5.50 R4.

### Co-immunoprecipitation (Co-IP)

Isolated glomerulus or HEK293 cells expressing CLDN5 with ZO1 and ZONAB were lysed in 50 mM Tris (pH 8.0) by 25–30 repeated passages through a 25-gauge needle, followed by centrifugation at 5000 × *g*. The membranes of lysis were extracted using CSK buffer (150 mM NaCl; 1% Triton X-100; 50 mM Tris, pH 8.0; and protease inhibitors). The membrane extract was precleared by incubation with protein A/G-sepharose (Sigma-Aldrich) prior to Co-IP. The precleared membrane extract was incubated for 16 h at 4 °C with anti-CLDN5, anti-ZO1, anti-ZONAB, and anti-mouse IgG (as negative control) antibodies. Antibody-bound material was pelleted with protein A/G-sepharose, washed three times with CSK buffer, and detected by immunoblotting.

### RNA immunoprecipitation (RIP)

To assess the association of endogenous ZONAB with endogenous Wif1 mRNA, immunoprecipitation (IP) of nuclear ribonucleoprotein (RNP) complexes was performed using the Imprint^®^ RNA Immunoprecipitation Kit (Sigma, USA). Isolated glomerulus lysates were incubated overnight at 4 °C with gentle rotation in the presence of excess (10 μg) IP antibody (IgG, anti-ZONAB). RNA in IP materials was used in RT reactions followed by qPCR analysis to detect the levels of Wif1 and β-actin mRNAs.

### Statistics and reproducibility

The significance of differences between groups was tested by Prism 6.07 (GraphPad Software Inc.). Statistical analysis was performed using two-tailed unpaired Student’s *t* test determine differences between two groups. *P*-values <0.05 were interpreted as statistically significant. All data are presented as mean ± SEM and other details such as the number of replicates and the level of significance are mentioned in figure legends and Supplementary Information/Source data file. The representative figures shown in the paper were repeated at least three independent experiments unless otherwise stated.

### Study approval

All animal studies were approved by the Animal Ethics Committee of Binzhou Medical University and conducted in accordance with the National Institutes of Health Guide for the Care and Use of Laboratory Animals.

### Reporting summary

Further information on research design is available in the Nature Research Reporting Summary linked to this article.

## Supplementary information

Supplementary Information

Description of Additional Supplementary Information

Reporting Summary

## Data Availability

All data generated in this study are provided in the Supplementary Information/Source data file. Exact *P* values are also included within the Source data file. The raw RNA-seq data generated in this study have been deposited in NCBI sequence read archive (SRA) database under accession number PRJNA700678. Additional details on protocols and databases/datasets used in this study are available from the corresponding author on reasonable request. Source data are provided with this paper.

## Source data

Source Data
