# Supplementary material for: Loss of CLDN5 in podocytes deregulates WIF1 to activate WNT signaling and contributes to kidney disease
Source: Nat Commun. 2022 Mar 24;13:1600. doi: 10.1038/s41467-022-29277-6 (PMC8948304; doi:10.1038/s41467-022-29277-6)
Supplement: Supplementary file 1 — Supplementary Information [file 41467_2022_29277_MOESM1_ESM.pdf]

Loss of CLDN5 in podocytes deregulates WIF1 to activate WNT signaling and contributes to kidney disease

Hui Sun[1, #], Hui Li[2, #], Jie Yan[1, #], Xiangdong Wang[1], Mengyuan Xu[1], Mingxia Wang[1], Baozhen Fan[1], Jieying Liu[1], Ninghua Lin[3], Xin Wang[4], Li Li[1], Shengtian Zhao[5, 6, 7, *], Yongfeng Gong[1, *]

1 Department of Physiology, Binzhou Medical University, 264003 Yantai, PR China
2 Department of Urology, West China Hospital, Sichuan University, 610041 Chengdu, PR China
3 Department of Anesthesiology, Binzhou Medical University, 264003 Yantai, PR China
4 Department of Pathophysiology, Qingdao University, 266071 Qingdao, PR China
5 Department of Urology, Binzhou Medical University Hospital, 256603 Yantai, PR China
6 Shandong Provincial Engineering Laboratory of Urologic Tissue Reconstruction, 250021 Jinan, PR China
7 Department of Urology, Shandong Provincial Hospital Affiliated to Shandong First Medical University, 250021 Jinan, PR China

[#] These three authors contributed equally to this work

* Corresponding author emails: ygong@bzmc.edu.cn, zhaoshengtian@sdu.edu.cn

Table of Contents

Supplementary Fig 1: Other CLDNs expression in podocyte-specific Cldn5 deletion mice.

Supplementary Fig 2: Podocyte genes expression in podocyte-specific Cldn5 deletion mice.

Supplementary Fig 3: Podocyte-specific Cldn5 deletion exacerbates podocyte injury in DN.

Supplementary Fig 4: Conditional knockout of Wif1 in podocytes induces a phenotype similar to that caused by knockout of Cldn5.

Supplementary Fig 5: Podocyte-specific loss of CLDN5 or WIF1 exacerbates interstitial fibrosis 14 days following the UUO operation.

Supplementary Fig 6: Disruption of WIF1 expression promotes UUO-induced renal fibrosis accompanied by up-regulated WNT downstream genes expression.

Supplementary Fig 7: Regulation of Wif1 expression by ZONAB.

Supplementary Fig 8: Summary.

Supplementary Table 1: Weight, 24-h urine output, and urine osmolality of WT and Cldn5 KO mice at indicated ages

Supplementary Table 2: Metabolic index

Supplementary Table 3: Primary antibody list

Supplementary Table 4: Secondary antibody list

Supplementary Table 5: Primer sequences used for qRT-PCR

Supplementary Information

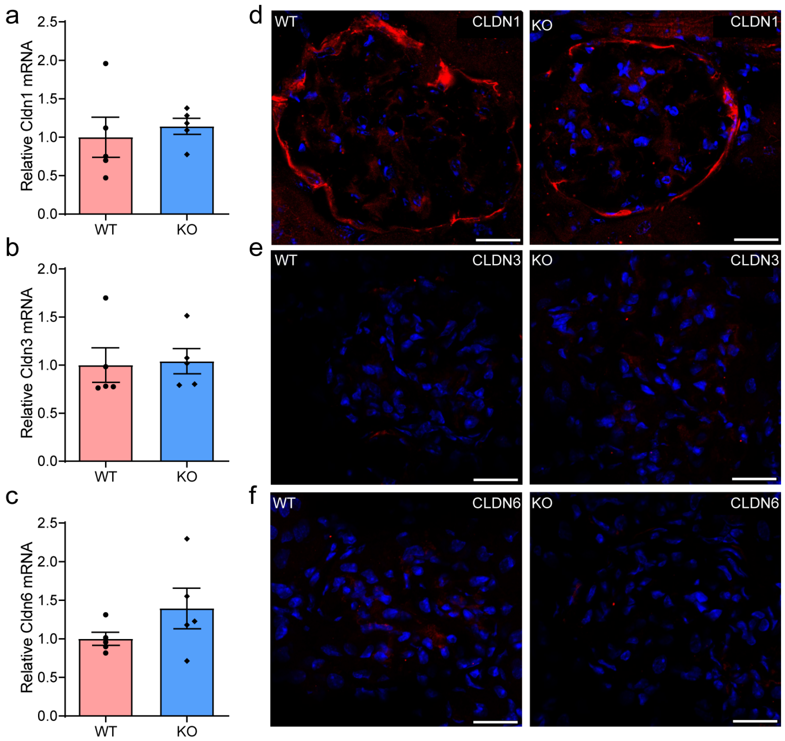

Supplementary Fig. 1. Other CLDNs expression in podocyte-specific Cldn5 deletion mice. (a-c) qRT-PCR analysis showing Cldn1 **(a)**, Cldn3 **(b)**, and Cldn6 **(c)** mRNA abundance in isolated glomerulus from WT and podocyte-specific Cldn5 KO mice (n = 5 biologically independent animals). Data are presented as mean values ± SEM. Two-tailed unpaired Student's t test was used for statistical comparisons. **(d-f)** Immunofluorescence staining showing the abundance and distribution pattern of CLDN1 **(d)**, CLDN3 **(e)**, and CLDN6 **(f)** in kidney sections of WT and podocyte-specific Cldn5 KO mice. Nuclei were visualized by DAPI. Scale bar, 20 μm. Source data are provided as a Source Data file.

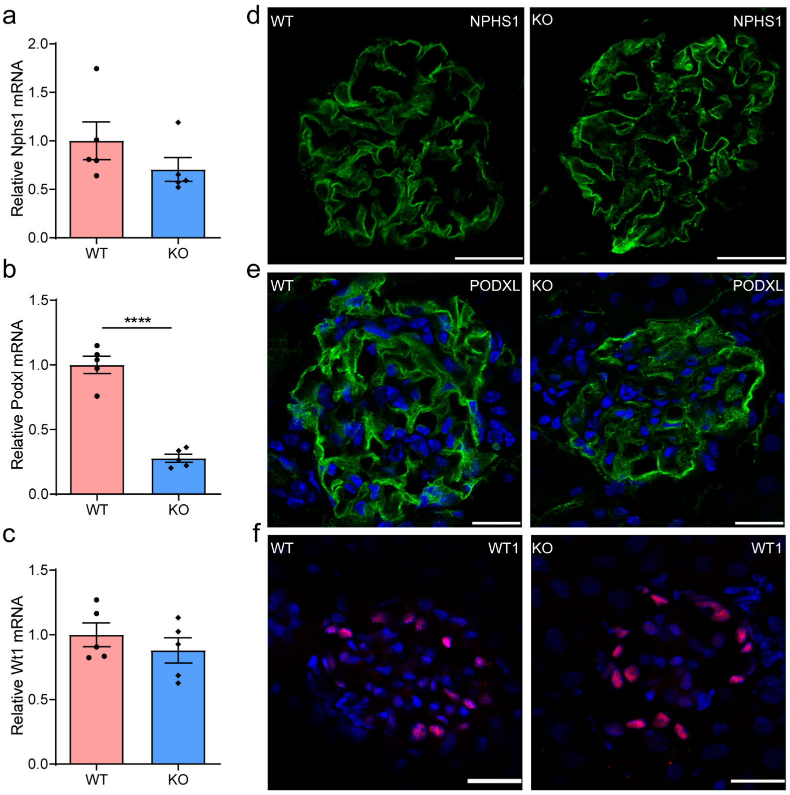

Supplementary Fig. 2. Podocyte genes expression in podocyte-specific Cldn5 deletion mice. (a-c) qRT-PCR analysis showing Nphs1 **(a)**, Podxl **(b)**, and Wt1 **(c)** mRNA abundance in isolated glomerulus from WT and podocyte-specific Cldn5 KO mice (n = 5 biologically independent animals, ****P < 0.0001). Data are presented as mean values ± SEM. Two-tailed unpaired Student's t test was used for statistical comparisons. **(d-f)** Immunofluorescence staining showing the abundance and distribution pattern of NPHS1 **(d)**, PODXL **(e)**, and WT1 **(f)** in kidney sections of WT and podocyte-specific Cldn5 KO mice. Nuclei were visualized by DAPI. Scale bar, 20 µm. Note that PODXL mRNA and staining signals are markedly reduced in Cldn5 KO mice. Source data are provided as a Source Data file.

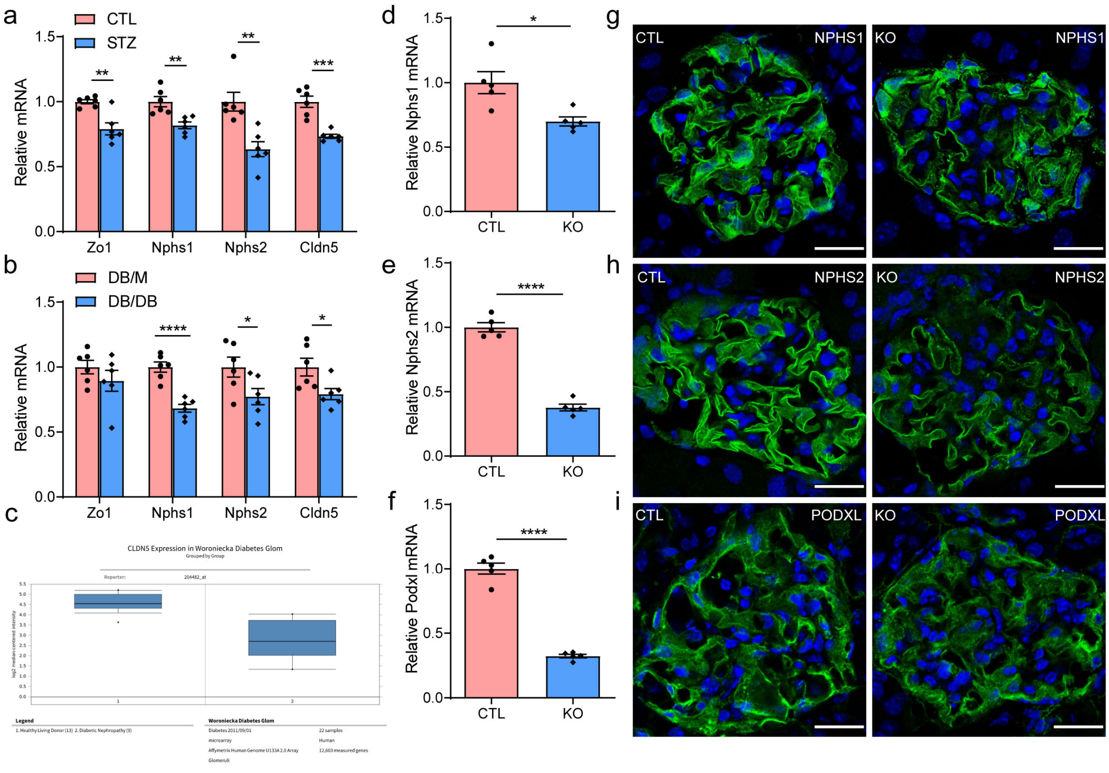

Supplementary Fig. 3. Podocyte-specific Cldn5 deletion exacerbates podocyte injury in DN. (a) mRNA expression of Zo1, Nphs1, Nphs2, and Cldn5 assessed by qRT–PCR in isolated glomerulus from control (CTL) and STZ-induced type I diabetic mice (STZ), 12 weeks after the last injection of STZ (n = 6 biologically independent animals, **P < 0.01, ***P < 0.001). **(b)** mRNA expression of Zo1, Nphs1, Nphs2, and Cldn5 assessed by qRT–PCR in isolated glomerulus from DB/M and DB/DB type 2 diabetic mice at 24 weeks of age (n = 6 biologically independent animals, *P < 0.05, ****P < 0.0001). **(c)** Nephroseq analysis comparing Cldn5 expression level in non-diabetic (n = 13) vs. diabetic individuals (n = 9) from Woroniecka diabetic glomerulus dataset. Whiskers represent the 10th/90th percentile values as precomputed within Nephroseq. The box represents the middle quartiles, the lines indicate the median, and the whiskers plots depict the maximum and minimum values of each group.

Nephroseq (www.nephroseq.org, 02/2021, University of Michigan, Ann Arbor, MI) was used for analysis and visualization. **(d-f)** mRNA expression of Nphs1 **(d)**, Nphs2 **(e)**, and Podxl **(f)** assessed by qRT–PCR in glomerulus from CTL and podocyte-specific Cldn5 KO diabetic mice (n = 5 biologically independent animals, *P < 0.05, ****P < 0.0001). **(g-i)** Representative confocal microscopic images showing the expressions of NPHS1 **(g)**, NPHS2 **(h)**, and PODXL **(i)** in kidney sections from CTL and podocyte-specific Cldn5 KO diabetic mice. Nuclei were visualized by DAPI. Scale bar, 20 μm. Data are presented as mean values ± SEM. Two-tailed unpaired Student's t test was used for statistical comparisons (**a-f**). Source data are provided as a Source Data file.

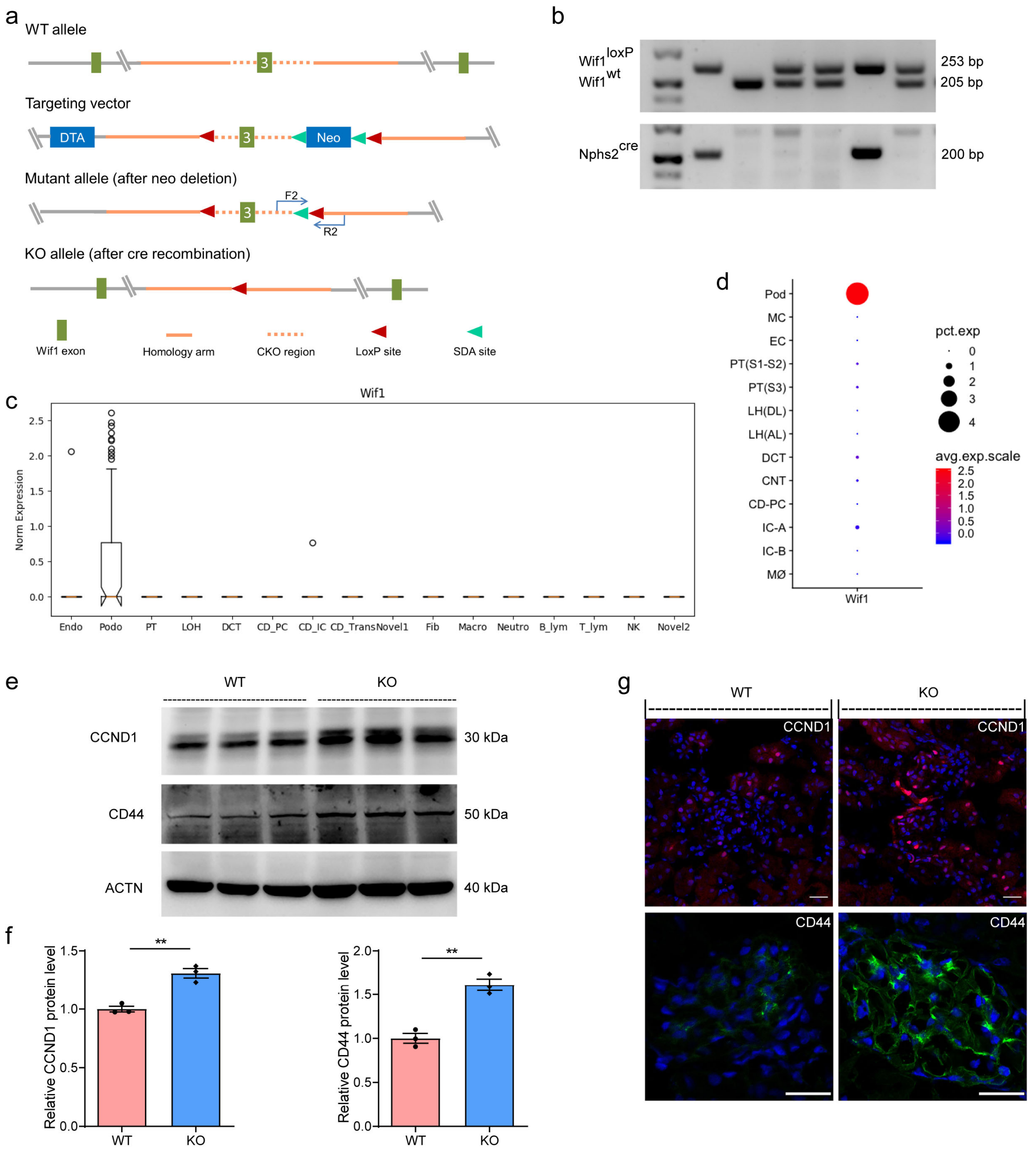

Supplementary Fig. 4. Conditional knockout of Wif1 in podocytes induces a phenotype similar to that caused by knockout of Cldn5. (a) Gene targeting strategy. The diagram showing the WT Wif1 locus, the targeting construct, the mutant allele after neo deletion, and the KO allele after Cre recombination. In the targeting vector, the neo cassette was flanked by SDA (self-deletion anchor) sites, and exon 3 of Wif1 was flanked by loxP sites. DTA was used for negative selection.

(b) PCR verification of derived mice tails, demonstrating WT (205 bp), heterozygous (205 bp and 253 bp), and homozygous (253 bp) alleles. The primer F2 is located on conditional knockout (cKO) region. The primer R2 is located downstream of the second loxP site. The Nphs2 Cre recombinase transgene was identified as a 200 bp PCR product. **(c-d)** Single-cell RNA sequencing data of Wif1 from Susztak lab's mouse kidney single cell atlas **(c)** and Humphreys lab's Kidney Interactive Transcriptomics **(d)**. **(e)** Immunoblotting for WNT target genes (CD44 and CCND1) in isolated glomerulus from WT and podocyte-specific Wif1 KO mice. **(f)** Bar graphs showing the fold changes in CD44 and CCND1 expression determined by densitometric analysis ($n = 3$ biologically independent animals, $^{**}P < 0.01$). Data are presented as mean values ± SEM. Two-tailed unpaired Student's t test was used for statistical comparisons. **(g)** Immunofluorescence staining for CCND1 and CD44 in kidney sections of WT and podocyte-specific Wif1 KO mice. Nuclei were visualized by DAPI. Scale bar, 20 μm. Source data are provided as a Source Data file.

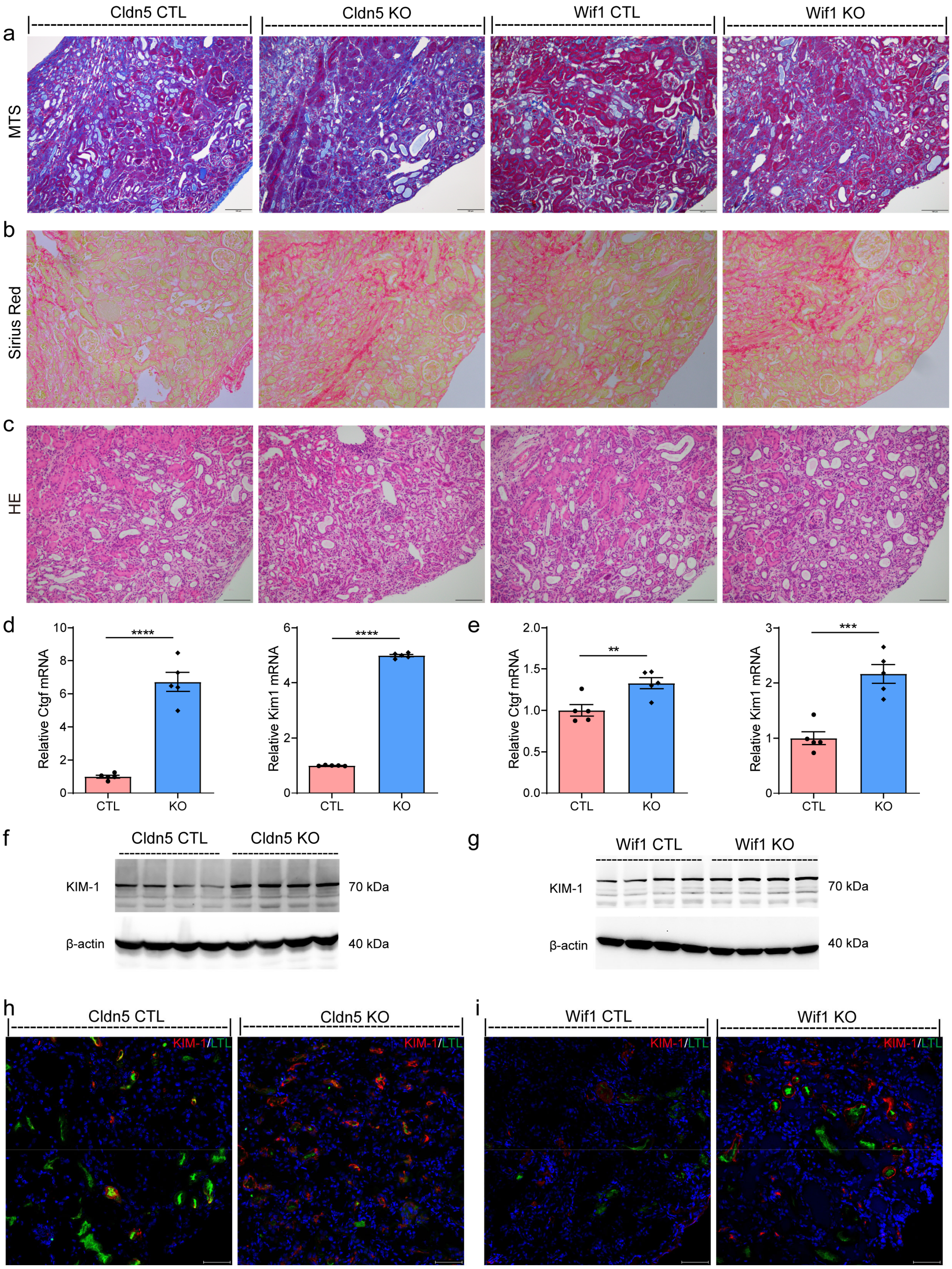

Supplementary Fig. 5. Podocyte-specific loss of CLDN5 or WIF1 exacerbates interstitial fibrosis 14 days following the UUO operation. (a-c) Representative microphotographs of Masson's trichrome staining (MTS) **(a)**, Sirius Red unpolarized

(b), and HE **(c)** staining of kidneys from the podocyte-specific Cldn5 KO and Wif1 KO mice compared to their respective control mice. Scale bars: 50 µm. **(d-e)** qRT-PCR analysis of Ctgf and Kim1 mRNA abundance in UUO-injured kidneys from the podocyte-specific Cldn5 KO mice **(d)** and Wif1 KO **(e)** mice compared to their respective control mice (n = 5 biologically independent animals, **P < 0.01, ***P < 0.001, ****P < 0.0001). Data are presented as mean values ± SEM. Two-tailed unpaired Student's t test was used for statistical comparisons. **(f-g)** Western blotting analysis of KIM-1 in UUO-injured kidneys from podocyte-specific Cldn5 KO **(f)** and Wif1 KO **(g)** mice compared to their respective control mice. **(h-i)** Immunofluorescen-ce for KIM-1 in kidney sections from the podocyte-specific Cldn5 KO **(f)** and Wif1 KO **(g)** mice compared to their respective control mice. LTL (green) was used as a proximal tubule marker. Scale bar, 20 µm. Nuclei were visualized by DAPI. Source data are provided as a Source Data file.

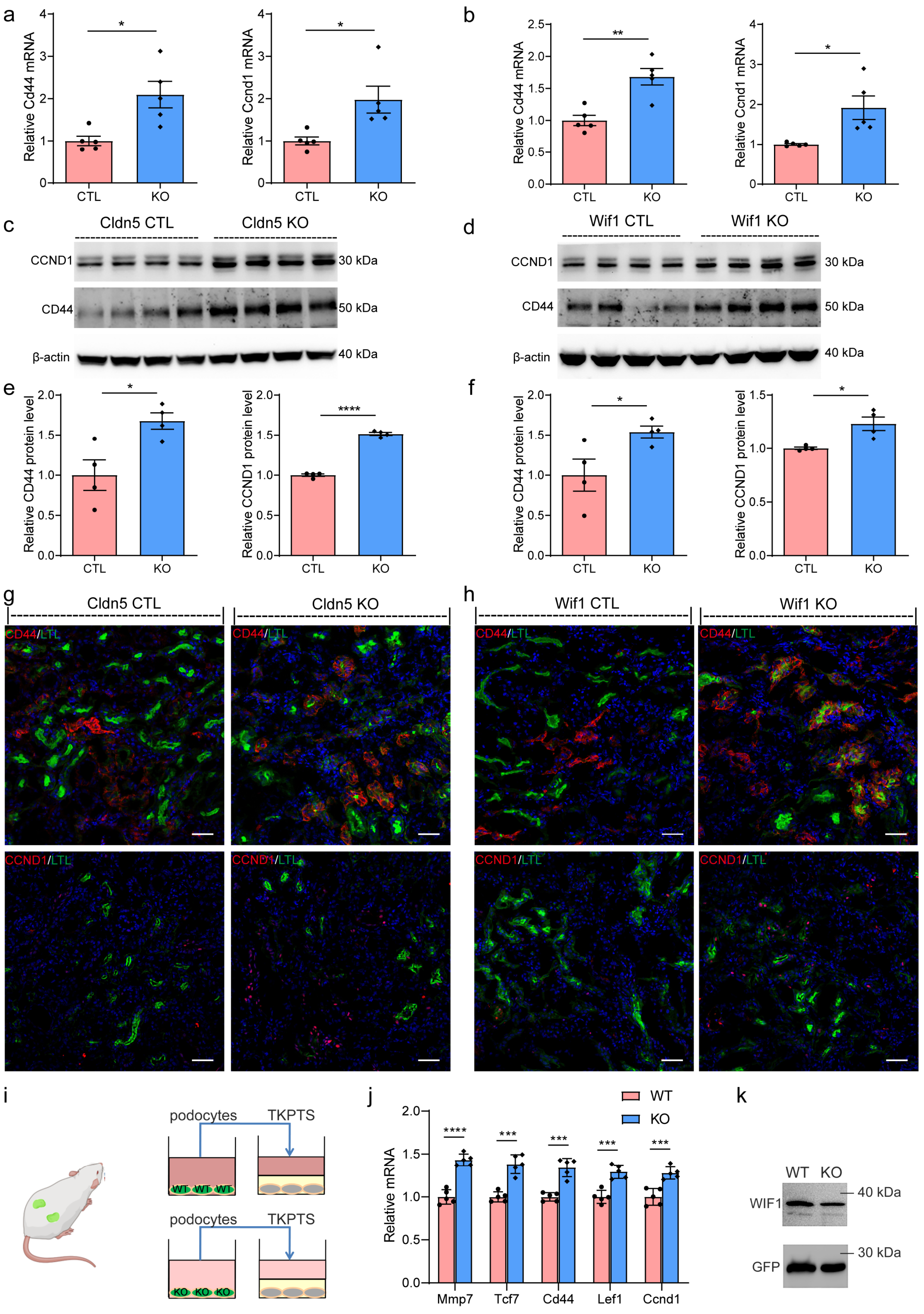

Supplementary Fig. 6. Disruption of WIF1 expression promotes UUO-induced renal fibrosis accompanied by up-regulated WNT downstream genes

expression. (**a-b**) qRT-PCR showing of Cd44 and Ccnd1 mRNA abundance in UUO-injured kidneys from the podocyte-specific Cldn5 KO **(a)** and Wif1 KO **(b)** mice compared to their respective control mice (n = 5 biologically independent animals, *P < 0.05, **P < 0.01). **(c-d)** Western blotting analysis of CD44 and CCND1 in UUO-injured kidneys from the podocyte-specific Cldn5 KO **(c)** and Wif1 KO **(d)** mice compared to their respective CTL mice. **(e-f)** Bar graphs (**e** for Cldn5 CTL and Cldn5 KO, **f** for Wif1 CTL and Wif1 KO) showing the mean ± SEM of fold changes in CD44 and CCND1 expression corresponding to densitometric analyses. Densitometry calculations from four samples/group are shown and are normalized to β-actin as indicated (n = 5 biologically independent animals, *P < 0.05, ****P < 0.0001). **(g-h)** Immunofluorescence for CD44 and CCND1 in kidney sections from the podocyte-specific Cldn5 KO **(g)** and Wif1 KO **(h)** mice compared to their respective control mice. Scale bar, 20 μm. LTL (green) was used as a proximal tubule marker. Nuclei were visualized by DAPI. (**i**) Schematic illustration of treatment of TKPTS with conditioned medium harvested from FACS sorted WT and Cldn5 KO podocytes. The mouse icon was created with BioRender.com. **(j)** WNT target genes Mmp7, Tcf7, Cd44, Lef1, and Ccnd1 mRNA expression in TKPTS cultured in conditioned medium (n = 5, ***P < 0.001, ****P < 0.0001). **(k)** Western blotting analysis of WIF1 in TKPTS medium. GFP served as an WIF1 expression reference. Data are presented as mean values ± SEM. Two-tailed unpaired Student's t test was used for statistical comparisons. (**a-b, e-f, j**). Source data are provided as a Source Data file.

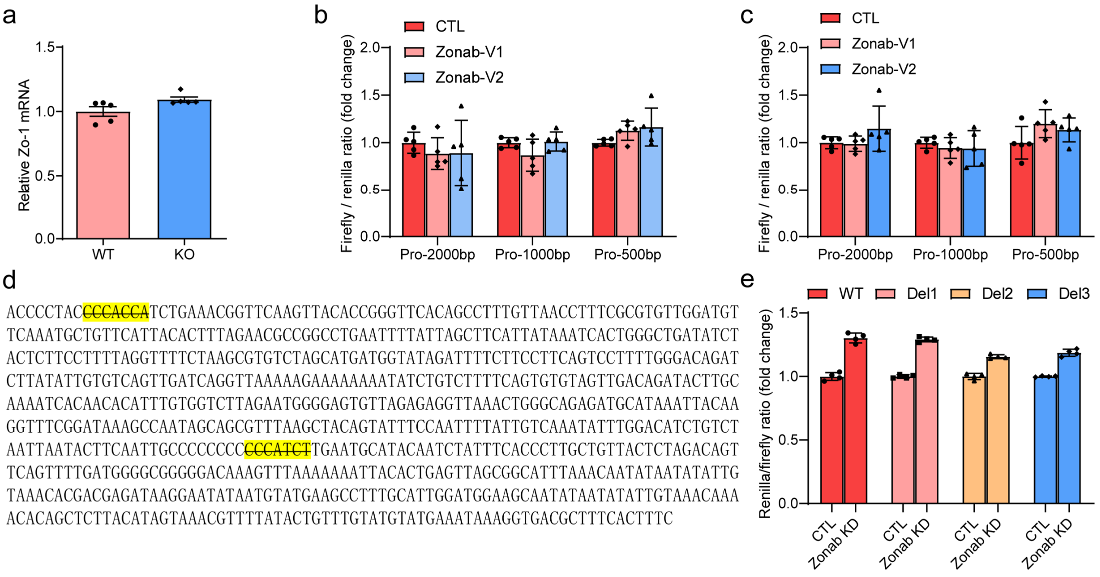

Supplementary Fig. 7. Regulation of Wif1 expression by ZONAB. (a) qRT-PCR analysis showing Zo1 mRNA abundance in isolated glomerulus from WT and podocyte-specific Cldn5 KO mice (n = 5 biologically independent animals,). **(b-c)** Luciferase reporter activity of WIF1 promoter fragments in MDCK **(b)** and primary podocytes **(c)** transfected with empty vector pCMV6 (CTL), pCMV6-Zonab-V1, or pCMV6-Zonab-V2 (n = 5 independent experiments). **(d)** 3'-UTR region of Wif1 mRNA with the predicted binding sites for ZONAB (highlight and delete line). **(e)** Renilla/firefly luciferase activity ratios measured in primary mouse podocytes transfected with siRNA for Zonab and constructs containing either WT or the indicated mutant 3'-UTRs (Del1 for 1st binding site deletion, Del2 for 2nd binding site deletion, and Del3 for both of the binding sites deletion) (n = 4 independent experiments). Data are presented as mean values ± SEM. Two-tailed unpaired Student's t test was used for statistical comparisons (**a-b, c, e**). Source data are provided as a Source Data file.

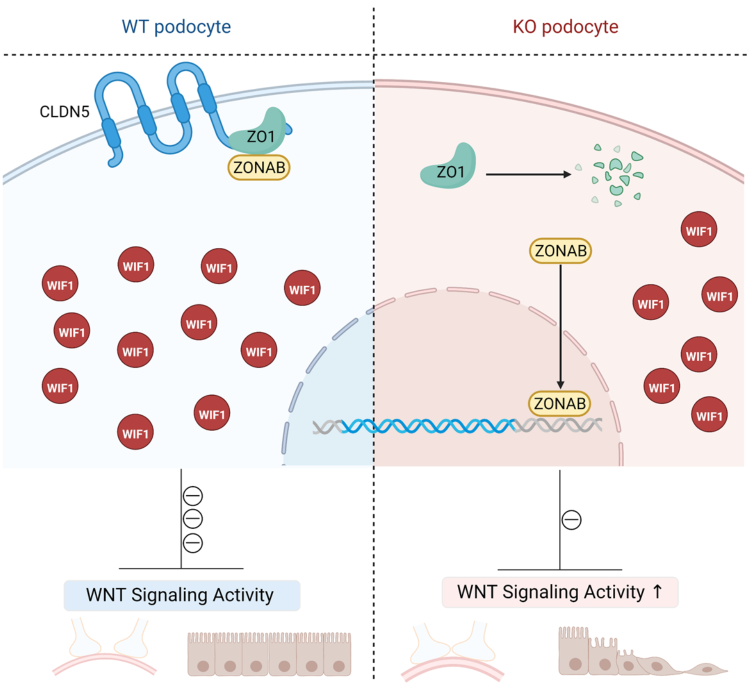

Supplementary Fig. 8. Summary. In WT podocytes under physiologic conditions, CLDN5 forms a complex with ZO1 and ZONAB, and this complex is required to sustain ZONAB's subcellular localization (left). Normal podocytes produce adequate level of WIF1 which negatively regulate WNT signaling activity in podocytes and tubular epithelial cells (left). CLDN5 absence reduces ZO1 expression and induces the nuclear translocation of transcription factor ZONAB, followed by transcriptional downregulation of Wif1, which decreases local (glomerular and tubular) WIF1 concentrations and leads to activation of WNT signaling pathway (right). The graphic was created with BioRender.com.

Supplementary Table 1: Weight, 24-h urine output, and urine osmolality of WT and Cldn5 KO mice at indicated ages

		Weight (g)	Urine Output (ml/24h)	Urine Osmolality (mOsm/kg）
**3W**	WT	14.17 ± 0.44	0.745 ± 0.025	1478.75 ± 106.08
	Cldn5 KO	13.76 ± 0.56	0.755 ± 0.041	1592.88 ± 163.11
**6W**	WT	21.23 ± 0.73	1.186 ± 0.036	1547.50 ± 101.36
	Cldn5 KO	21.99 ± 0.60	1.206 ± 0.055	1662.13 ± 179.64
**12W**	WT	30.12 ± 0.77	1.610 ± 0.062	1367.63 ± 97.65
	Cldn5 KO	30.48 ± 0.36	1.678 ± 0.057	1527.25 ± 191.49
**24W**	WT	34.04 ± 1.12	1.830 ± 0.098	1532.25 ± 196.79
	Cldn5 KO	36.32 ± 1.89	1.926 ± 0.100	1684.50 ± 208.45
**48W**	WT	37.60 ± 0.65	1.976 ± 0.042	1442.88 ± 165.58
	Cldn5 KO	39.63 ± 2.80	2.054 ± 0.113	1529.25 ± 196.98

Data are presented as mean values ± SEM. n = 10 biologically independent animals for each group.

Supplementary Table 2: Metabolic index

	**Cldn5-CTL**	**Cldn5-KO**	**Wif1-CTL**	**Wif1-KO**
**Weight (g)**	19.23 ± 1.13	21.58 ± 1.45	20.17 ± 1.63	20.31 ± 1.22
**Blood glucose (g/dL)**	31.28 ± 2.15	28.53 ± 3.84	30.68 ± 3.65	31.89 ± 2.21
**HbA1c (100%）**	15.43 ± 1.84	15.06 ± 2.58	16.11 ± 1.85	16.82 ± 2.40
**Food intake (g/day)**	3.30 ± 0.50	3.75 ± 0.88	3.67 ± 0.51	3.31 ± 0.59
**Water intake (g/day)**	20.90 ± 5.37	20.20 ± 4.87	21.50 ± 4.29	19.71 ± 4.17
**Urine Output (ml/day)**	23.00 ± 5.40	23.55 ± 5.81	21.10 ± 4.23	20.94 ± 4.33

Data are presented as mean values ± SEM. n = 10 biologically independent animals for each group.

Supplementary Table 3: Primary antibody list

Target	Product name	Host	Applic-ations	Dilution ratio	Cat#	Company
CLDN5	Claudin 5 Monoclonal Antibody (4C3C2)	Mouse	IF	100	35-2500	Invitrogen
			WB	1000		
CLDN5	Claudin 5 Polyclonal Antibody	Rabbit	WB	1000	34-1600	Invitrogen
			CoIP	50		
NPHS1	Mouse Nephrin Antibody	Goat	IF	200	AF3159	R&D systems
			WB	1000		
NPHS2	Anti-NPHS2 antibody	Rabbit	IF	400	ab50339	Abcam
PODXL	Mouse Podocalyxin Antibody	Goat	IF	100	AF1556	R&D systems
WT1	Recombinant Anti-Wilms Tumor Protein antibody [CAN-R9(IHC)-56-2]	Rabbit	IF	100	ab89901	Abcam
ZO1	ZO-1 Polyclonal Antibody (ZMD.437)	Rabbit	IF	200	40-2300	Invitrogen
			CoIP	50		
ZO1	ZO-1 Monoclonal Antibody (ZO1-1A12)	Mouse	WB	1000	33-9100	Invitrogen
			CoIP	50		
ZONAB	ZONAB antibody	Rabbit	IF	200	A303-070A	BETHYL
ZONAB	ZONAB Polyclonal Antibody	Rabbit	WB	1000	40-2800	Invitrogen
			CoIP	50		
ZONAB	DBPA Antibody (4D9)	Mouse	WB	1000	H00008531-M02	Abnova
			CoIP	50		
WIF1	WIF1 Polyclonal Antibody	Rabbit	IF	100	PA5-76731	Invitrogen
WIF1	WIF1 Monoclonal Antibody (1G5)	Mouse	WB	1000	MA5-15701	Invitrogen
CTNNB1	Anti-Active-β-Catenin (Anti-ABC) Antibody, clone 8E7	Mouse	IF	50	05-665	Millipore
CD44	Purified anti-mouse/human CD44 Antibody, IM7	Rat	IF	100	103002	BioLegend
			WB	500		
Collagen I	Goat Anti-Type I Collagen-UNLB	Goat	IF	200	1310-01	Southern Biotech
Desmin	Anti-Desmin antibody	Rabbit	IF	300	ab15200	Abcam
α-SMA	Anti-alpha smooth muscle Actin antibody	Rabbit	IF	200	ab5694	Abcam
CLDN1	Claudin 1 Polyclonal Antibody	Rabbit	IF	100	PA5-32350	Invitrogen
CLDN3	Claudin-3 (D7A3O) Rabbit mAb	Rabbit	IF	100	83609	CST
CLDN6	Claudin-6 (E2S5M) Rabbit mAb	Rabbit	IF	100	62831	CST
GAPDH	Anti-GAPDH antibody [6C5]	Mouse	WB	1000	ab8245	Abcam
β-tubulin	beta Tubulin Monoclonal Antibody	Mouse	WB	1000	MA5-11732	Invitrogen

	(TBN06 (Tub 2.5))					
β-actin	Anti-beta Actin antibody	Mouse	WB	2000	ab8226	Abcam
β-actin	HRP-conjugated β-Actin mAb	Rabbit	WB	1000	AC028	Abclonal
Histone H3	Histone H3 (D1H2) XP® Rabbit mAb	Rabbit	WB	1000	4499	CST
CCND1	Cyclin D1 (E3P5S) XP® Rabbit mAb	Rabbit	IF	50	55506	CST
			WB	1000		
KIM-1	Rat TIM-1/KIM-1/HAVCR Antibody	Goat	IF	100	AF3689	R&D systems
GFP	GFP (D5.1) Rabbit mAb	Rabbit	WB	1000	2956	CST
VEGFA	Recombinant Anti-VEGFA antibody [EP1176Y] - C-terminal	Rabbit	IF	100	ab52917	Abcam
			WB	1000		
Phospho-Smad1/5/9	Phospho-Smad1 (Ser463/465)/ Smad5 (Ser463/465)/ Smad9 (Ser465/467) (D5B10) mAb	Rabbit	IF	100	13820	CST
			WB	1000		
FN	Anti-Fibronectin antibody	Rabbit	IF	200	ab2413	Abcam
			WB	1000		

Supplementary Table 4: Secondary antibody list

Product name	Conjugate	Applic -ations	Dilution ratio	Cat#	Company
Donkey Anti-Goat IgG Antibody	FITC conjugate	IF	200	AP180F	Millipore
Donkey Anti-Mouse IgG Antibody	Rhodamine conjugate	IF	200	AP192R	Millipore
Goat Anti-Mouse IgG Antibody	Rhodamine conjugate	IF	200	AP124R	Millipore
Goat Anti-Rabbit IgG Antibody	Rhodamine conjugate	IF	200	AP132R	Millipore
Goat Anti-Rabbit IgG Antibody	FITC conjugate	IF	200	AP132F	Millipore
Donkey Anti-Mouse IgG Antibody	FITC conjugate	IF	200	AP192F	Millipore
Goat Anti-Rat IgG Antibody	Rhodamine conjugate	IF	200	AP136R	Millipore
Donkey Anti-Rat IgG Antibody	FITC conjugate	IF	200	AP189F	Millipore
Goat Anti-Guinea Pig IgG Antibody	FITC conjugate	IF	200	AP108F	Millipore
Goat Anti-Mouse IgG Antibody	FITC conjugate	IF	200	AP124R	Millipore
Goat anti-Mouse IgG (H+L) Secondary Antibody	HRP	WB	10000	31430	ThermoFish er
Goat anti-Rabbit IgG (H+L) Secondary Antibody	HRP	WB	10000	31460	ThermoFish er
Rabbit Anti-Goat IgG (H+L) Secondary Antibody	HRP	WB	10000	S0010	Affinity

Supplementary Table 5: Primer sequences used for qRT-PCR

Gene	Forward	Reverse
Cldn5	GTTAAGGCACGGGTAGCACT	GTACTTCTGTGACACCGGCA
Nphs1	CAGCTGCTAGTCTGCGAGG	ATCAATGACAGGAGGTCCTG
Nphs2	GTGAGGAGGGCACGGAAGT	TAATCCAGAGGGCTTGATGC
Podxl	GAAGCCCACTACACACAAACC	ACACAGCAGTTCCACGAGTT
Wt1	TTCACCTTGCACTTCTCGGG	TGACCGTGCTGTATCCTTGG
Wif1	GTGAACTCAGCAAATGCCCC	CTCTCGACACTGGCACTTGT
Zo1	GCCGCTAAGAGCACAGCAA	TCCCCACTCTGAAAATGAGGA
Cldn1	TGGGGCTGATCGCAATCTTT	CACTAATGTCGCCAGACCTGA
Cldn3	ACTGCGTACAAGACGAGACG	GTAGTCCTTGCGGTCGTAGG
Cldn6	GTCTCTTTTGCAGGCTCGGA	CGATGCTGTTGCCGATGAAG
Cd44	GAAGACGAAAACCATCCAACAACT	CATCTTTAGCGCCGCTCTTAG
Lef1	CCATCACGGGTGGATTCAG	CATATGATGGGAAAACCTGGACAT
Tcf7	CAACCCCCGCTGCATAAC	GAAATGTTCGTAGAGTGGAGAAAGC
Mmp7	TTTGCTGCCACCCATGAAT	CTCTTTGATAGGTAGGGTACATCACAGT
Ccnd1	GCGTACCCTGACACCAATCTC	CTCCTCTTCGCACTTCTGCTC
Actb	GGCTGTATTCCCCTCCATCG	CCAGTTGGTAACAATGCCATGT
Cd44-V1	CACCATTGCCTCAACTGTGC	TTGTGGGCTCCTGAGTCTGA
Cd44-V2	CACCTTGGCCACCACTCCTAA	TCTTTAGCGCCGCTCTTAGT
Cd44-V3	TCGATTTGAATGTAACCTGCCG	CAGTCCGGGAGATACTGTAGC
Cd44-V4	CTTCAATGCCTCAGCCCCTC	GTACTTGCAATGGTGGCCAAG
Cd44-V5	AGCCCCTCCTGAAGAAGACT	GACTGGAGTCTGTATTGGTGGC
Cd44-V6	CAACCGTGATGGTACTCGCT	AGAATGACTCTGTGTGGTGGC
Kim1	CTGGAATGGCACTGTGACATCC	GCAGATGCCAACATAGAAGCCC
Col1a1	CCTCAGGGTATTGCTGGACAAC	CAGAAGGACCTTGTTTGCCAGG
Ctgf	TGCGAAGCTGACCTGGAGGAAA	CCGCAGAACTTAGCCCTGTATG
Fn	CCCTATCTCTGATACCGTTGTCC	TGCCGCAACTACTGTGATTCGG
