# Supplementary material for: Loss of CLDN5 in podocytes deregulates WIF1 to activate WNT signaling and contributes to kidney disease
Source: Nat Commun. 2022 Mar 24;13:1600. doi: 10.1038/s41467-022-29277-6 (PMC8948304; doi:10.1038/s41467-022-29277-6)
Supplement: Supplementary file 2 — Description of Additional Supplementary Information [file 41467_2022_29277_MOESM2_ESM.pdf]

Additional Description of Supplementary Information

Editorial-policy-checklist

Author checklist

Supplementary Information

Reporting Summary
