# Supplementary material for: Loss of CLDN5 in podocytes deregulates WIF1 to activate WNT signaling and contributes to kidney disease
Source: Nat Commun. 2022 Mar 24;13:1600. doi: 10.1038/s41467-022-29277-6 (PMC8948304; doi:10.1038/s41467-022-29277-6)
Supplement: Supplementary file 3 — Reporting Summary [file 41467_2022_29277_MOESM3_ESM.pdf]

nature research

Corresponding author(s): Yongfeng Gong

Last updated by author(s): Feb 14, 2022

Reporting Summary

Nature Research wishes to improve the reproducibility of the work that we publish. This form provides structure for consistency and transparency in reporting. For further information on Nature Research policies, see our Editorial Policies and the Editorial Policy Checklist.

Statistics

For all statistical analyses, confirm that the following items are present in the figure legend, table legend, main text, or Methods section.

n/a	Confirmed	
☐	☒	The exact sample size (*n*) for each experimental group/condition, given as a discrete number and unit of measurement
☐	☒	A statement on whether measurements were taken from distinct samples or whether the same sample was measured repeatedly
☐	☒	The statistical test(s) used AND whether they are one- or two-sided<br>*Only common tests should be described solely by name; describe more complex techniques in the Methods section.*
☐	☒	A description of all covariates tested
☐	☒	A description of any assumptions or corrections, such as tests of normality and adjustment for multiple comparisons
☐	☒	A full description of the statistical parameters including central tendency (e.g. means) or other basic estimates (e.g. regression coefficient) AND variation (e.g. standard deviation) or associated estimates of uncertainty (e.g. confidence intervals)
☐	☒	For null hypothesis testing, the test statistic (e.g. F, t, r) with confidence intervals, effect sizes, degrees of freedom and P value noted<br>*Give P values as exact values whenever suitable.*
☒	☐	For Bayesian analysis, information on the choice of priors and Markov chain Monte Carlo settings
☒	☐	For hierarchical and complex designs, identification of the appropriate level for tests and full reporting of outcomes
☒	☐	Estimates of effect sizes (e.g. Cohen's d, Pearson's r), indicating how they were calculated

Our web collection on statistics for biologists contains articles on many of the points above.

Software and code

Policy information about availability of computer code

Data collection	Histology images were collected by cellSens Software v3.1. Transmission electron microscopy images were collected by RADIUS Software v2.1. Immunofluorescence images were collected and processed by Zen Software v2.3 (blue edition). Luciferase activities were collected by Omega Software v5.50 R4. qRT-PCR results were acquired by QuantStudioTM Design & Analysis SE Software v1.5.0.
Data analysis	All figures integration were performed on Adobe Illustrator 2021. Statistical analyses were performed using GraphPad Prism 6.07; Image J v1.8.0 was used to quantify Western blotting results and Periodic Acid-Schiff staining micrographs. Paired-end RNA-seq clean reads were aligned to the mouse reference genome (Ensemble_GRCm38.90) with TopHat (version 2.0.12), and the aligned reads were used to quantify mRNA expression by using HTSeq-count (version 0.6.1). Differential expression analysis of two groups was performed using the DESeq2 R package (1.16.1). The resulting P-values were adjusted using the Benjamini and Hochberg's approach for controlling the false discovery rate.

For manuscripts utilizing custom algorithms or software that are central to the research but not yet described in published literature, software must be made available to editors and reviewers. We strongly encourage code deposition in a community repository (e.g. GitHub). See the Nature Research guidelines for submitting code & software for further information.

Data

Policy information about availability of data

All manuscripts must include a data availability statement. This statement should provide the following information, where applicable:

- Accession codes, unique identifiers, or web links for publicly available datasets
- A list of figures that have associated raw data
- A description of any restrictions on data availability

The raw RNA-seq data were deposited in NCBI sequence read archive (SRA) database, with the accession number PRJNA700678 (https://www.ncbi.nlm.nih.gov/Traces/study/?acc=PRJNA700678). This data has been publicly released on 02/12/2022. Paired-end RNA-seq clean reads were aligned to the mouse reference

genome (Ensemble_GRCm38.90) . The source image for Supplementary Fig 3c may be found at Nephroseq database (nephroseq.org). The source images for Supplementary Fig 4c was obtained from Susztak Lab Kidney Biobank multi-omics datasets (https://susztaklab.com/developing_adult_kidney/scRNA/). The source images for Supplementary Fig 4d was obtained from Humphreys lab Kidney Interactive Transcriptomics (http://www.humphreyslab.com/SingleCell/displaycharts.php). Additional source data can be obtained by contacting the lead author.

Field-specific reporting

Please select the one below that is the best fit for your research. If you are not sure, read the appropriate sections before making your selection.

☒ Life sciences ☐ Behavioural & social sciences ☐ Ecological, evolutionary & environmental sciences

For a reference copy of the document with all sections, see nature.com/documents/nr-reporting-summary-flat.pdf

Life sciences study design

All studies must disclose on these points even when the disclosure is negative.

Sample size

Sample sizes were chosen based on preliminary data and suggested a large effect size. The sample size for diabetic model were at least 10 animals per group. The sample size for UUO model were at least 7 animals per group. These sample sizes were thought to be statistically sufficient based on literatures involving DN and UUO. The sample size for knockout phenotype analysis was 3-5 animals per group according to our paper published, such as J Am Soc Nephrol. 2017 Jan;28(1):106-117. In vitro studies were performed in at least three replicated according to our paper published, such as EMBO J. 2012 Apr 18;31(8):1999-2012. Sample sizes were indicated in the legend of each Figure and Supplementary Figure.

Data exclusions

No data were excluded from the analyses.

Replication

Each experiment was repeated three times under same conditions and all attempts at replication were successful.

Randomization

Mice were assigned to each group randomly. The cells in vitro from each cell line were pooled, and then seeded and treated randomly.

Blinding

Phenotyping in mouse was performed blinded (the investigator had no knowledge of the genotype at that point in time). For in vivo experiments, the investigators were blinded to group allocation during data collection and analysis. Sample preparing such as animal AAV treatment, cells culture and treatment was conducted by one person. Data collecting and analysis of Western blotting, qRT-PCR, immunofluorescence were performed by another person blindly.

Reporting for specific materials, systems and methods

We require information from authors about some types of materials, experimental systems and methods used in many studies. Here, indicate whether each material, system or method listed is relevant to your study. If you are not sure if a list item applies to your research, read the appropriate section before selecting a response.

Materials & experimental systems

n/a	Involved in the study	
☐	☒	Antibodies
☐	☒	Eukaryotic cell lines
☒	☐	Palaeontology and archaeology
☐	☒	Animals and other organisms
☒	☐	Human research participants
☒	☐	Clinical data
☒	☐	Dual use research of concern

Methods

n/a	Involved in the study	
☒	☐	ChIP-seq
☒	☐	Flow cytometry
☒	☐	MRI-based neuroimaging

Antibodies

Antibodies used

Anti-Claudin 5 antibody, CoIP, IF, WB, Invitrogen, 35-2500, 4C3C2, UB280529; Anti-Claudin 5 antibody, CoIP, Invitrogen, 34-1600, SJ259075; Anti-NPHS1 antibody, IF, WB, R&D, systems, AF3159, CBIK0318032; Anti-NPHS2 antibody, IF, Abcam, ab50339, GR3213171-1; Anti-PODXL antibody, IF, R&D, systems, AF1556, JPC0117081; Anti-WT1 antibody, IF, Abcam, ab89901, CAN-R9, (IHC)-56-2, GR3270281-5; Anti-ZO1 antibody, IF, WB, CoIP, Invitrogen, 40-2300, ZMD.437, UK287728; Anti-ZO1 antibody, IF, WB, CoIP, Invitrogen, 33-9100, VJ307393; Anti-ZONAB antibody, IF, BETHYL, A303-070A, A303-070A-1; Anti-ZONAB antibody, CoIP, WB, Invitrogen, 40-2800, UB277621; Anti-ZONAB antibody, CoIP, Abnova, H00008531-M02, 4D9, F4021-4D9; Anti-WIF1 antibody, IF, Invitrogen, PA5-76731, 311F6A27; Anti-WIF1 antibody, WB, Invitrogen, MA5-15701, 1G5, AA9817N; Anti-Ative-β-Catenin antibody, IF, Millipore, 05-665, 8E7, 3173793; Anti-CD44 antibody, IF, WB, BioLegend, 103002, IM7, B246298; Anti-Type I Collagen antibody, IF, WB, SouthernBiotech, 1310-01, B2918-TD09; Anti-Desmin antibody, IF, Abcam, ab15200, GR280024-10; Anti-alpha smooth muscle Actin antibody, IF, WB, Abcam, ab5694, GR3183259-27; Anti-Claudin1 antibody, IF, Invitrogen, PA5-32350, TK2659695; Anti-Claudin 3 antibody, IF, Cell Signaling Technology, 83609, D7A3O, 1; Anti-Claudin 6 antibody, IF, Cell Signaling Technology, 62831, E2S5M, 1; Anti-GAPDH antibody, WB, Affinity, AF7021, 62u0922; Anti-beta Tubulin antibody, WB, Invitrogen, MA5-11732, TBN06, (Tub2.5), TC253C541; Anti-Histone H3 antibody, WB, Cell Signaling Technology, 4499, D1H2, 9; Anti-Cyclin D1 antibody, IF, WB, Cell Signaling

Technology, 55506, E3P5S, 3; Anti-rTIM-1 antibody, IF, WB, R&D, systems, AF3689, XWS0618121; Anti-GFP, WB, Cell Signaling Technology, 2956, D5.1, 1; Recombinant Anti-VEGFA antibody [EP1176Y] - C-terminal, IF, WB, Abcam, ab52917, EP1176Y, Phospho-Smad1 (Ser463/465)/ Smad5 (Ser463/465)/ Smad9 (Ser465/467) (D5B10) Rabbit mAb, IF, WB, Cell Signaling Technology, 13820, Lot 3; Anti-Fibronectin antibody, IF, WB, Abcam, ab2413, GR270934-1; Anti-beta Actin antibody, WB, Abcam, ab8226, GR3206282-2; HRP-conjugated β-Actin Rabbit mAb, ABclonal, AC028, 9100026012; Donkey Anti-Goat IgG Antibody, FITC conjugate, IF, Millipore, AP180F, 2965870; Donkey Anti-Mouse IgG Antibody, Rhodamine conjugate, IF, Millipore, AP192R, 3114554; Goat Anti-Mouse IgG Antibody, Rhodamine conjugate, IF, Millipore, AP124R, 3084151; Goat Anti-Rabbit IgG Antibody, Rhodamine conjugate, IF, Millipore, AP132R, 2919757; Goat Anti-Rabbit IgG Antibody, FITC conjugate, IF, Millipore, AP132F, 2934662; Donkey Anti-Mouse IgG Antibody, FITC conjugate, IF, Millipore, AP192F, 3094628; Goat Anti-Rat IgG Antibody, Rhodamine conjugate, IF, Millipore, AP136R, 3070837; Donkey Anti-Rat IgG Antibody, FITC conjugate, IF, Millipore, AP189F, 3043071; Goat Anti-Guinea Pig IgG Antibody, FITC conjugate, IF, Millipore, AP108F, 3105304; Goat Anti-Mouse IgG Antibody, (H+L) FITC Conjugated, IF, Millipore, AP124R, 2946033; Goat anti-Mouse IgG (H+L) Secondary Antibody, HRP, WB, ThermoFisher, 31430, TG267017; Goat anti-Rabbit IgG (H+L) Secondary Antibody, HRP, WB, ThermoFisher, 31460, TG266717; Rabbit Anti-Goat IgG (H+L) HRP, WB, Affinity, S0010, 55g7583.

Validation

All antibodies are listed in Supplementary Table 2 including catalog numbers. Anti-Claudin 5 antibody and Anti-WIF1 antibody were validated by knockout mouse model in this study. The specific claudin-5 or WIF1 antibody yielded no signal when tested in a podocyte-specific knockout glomerulus while giving the specific target protein signal in the wild-type podocyte as determined by immunofluorescence. The expected band at the correct MW disappears in the knockout isolated glomeurlus as determined by Western blotting .; Anti-Ative-β-Catenin antibody reference: Wnt/β-catenin pathway in podocytes integrates cell adhesion, differentiation, and survival. Kato H, Gruenwald A, Suh JH, Miner JH, Barisoni-Thomas L, Taketo MM, Faul C, Millar SE, Holzman LB, Susztak K. J Biol Chem. 2011 Jul 22;286(29):26003-15.; Anti-Type I Collagen antibody and Anti-rTIM-1 antibody reference: Chronic epithelial kidney injury molecule-1 expression causes murine kidney fibrosis. Humphreys BD, Xu F, Sabbisetti V, Grgic I, Movahedi Naini S, Wang N, Chen G, Xiao S, Patel D, Henderson JM, Ichimura T, Mou S, Soeung S, McMahon AP, Kuchroo VK, Bonventre JV. J Clin Invest. 2013 Sep;123(9):4023-35.; Anti-alpha smooth muscle Actin antibody, Anti-Desmin antibody, Anti-rTIM-1 antibody, Anti-NPHS1 antibody, Anti-NPHS2 antibody, and Anti-WT1 antibody reference: Albumin contributes to kidney disease progression in Alport syndrome. Jarad G, Knutsen RH, Mecham RP, Miner JH. Am J Physiol Renal Physiol. 2016 Jul 1;311(1): F120-30.; Anti-PODXL antibody, Anti-ZO1 antibody, Anti-ZONAB antibody, Anti-CD44 antibody, Anti-Claudin1 antibody, Anti-Claudin 3 antibody, Anti-Claudin 6 antibody, Anti-GAPDH antibody, Anti-beta Tubulin antibody, Anti-Histone H3(D1H2) antibody, Anti-Cyclin D1 antibody, Anti-GFP, Anti-beta Actin antibody, Recombinant Anti-VEGFA antibody [EP1176Y] - C-terminal, Phospho-Smad1 (Ser463/465)/ Smad5 (Ser463/465)/ Smad9 (Ser465/467) (D5B10) Rabbit mAb, and Anti-Fibronectin antibody were all validated by the company.
1. Anti-PODXL antibody, IF, R&D, systems, AF1556, JPC0117081; https://www.rndsystems.com/cn/products/mouse-podocalyxin-antibody_af1556
2. Anti-ZO1 antibody, IF, WB, CoIP, Invitrogen, 40-2300, ZMD.437, UK287728; https://www.thermofisher.cn/cn/zh/antibody/product/ZO-1-Antibody-clone-ZMD-437-Polyclonal/40-2300
3. Anti-ZO1 antibody, IF, WB, CoIP, Invitrogen, 33-9100, VJ307393; https://www.thermofisher.cn/cn/zh/antibody/product/ZO-1-Antibody-clone-ZO1-1A12-Monoclonal/33-9100
4. Anti-ZONAB antibody, IF, BETHYL, A303-070A, A303-070A-1; https://www.thermofisher.cn/cn/zh/antibody/product/ZONAB-Antibody-Polyclonal/A303-070A
5. Anti-ZONAB antibody, WB, Invitrogen, 40-2800, UB277621; https://www.thermofisher.cn/cn/zh/antibody/product/ZONAB-Antibody-Polyclonal/40-2800
6. Anti-ZONAB antibody, CoIP, Abnova, H00008531-M02, 4D9, F4021-4D9;
7. Anti-CD44 antibody, IF, WB, BioLegend, 103002, IM7, B246298; https://www.biolegend.com/en-us/products/purified-anti-mouse-human-cd44-antibody-318
8. Anti-Claudin1 antibody, IF, Invitrogen, PA5-32350, TK2659695; https://www.thermofisher.cn/cn/zh/antibody/product/Claudin-1-Antibody-Polyclonal/PA5-32350
9. Anti-Claudin3 antibody, IF, Cell Signaling Technology, 83609, D7A3O, 1; https://www.cellsignal.cn/products/primary-antibodies/claudin-3-d7a3o-rabbit-mab/83609?site-search-type=Products&N=4294956287&Ntt=83609&fromPage=plp&_requestid=3329122
10. Anti-Claudin 6 antibody, IF, Cell Signaling Technology, 62831, E2S5M, 1; https://www.cellsignal.cn/products/primary-antibodies/claudin-6-e2s5m-rabbit-mab/62831?site-search-type=Products&N=4294956287&Ntt=62831&fromPage=plp&_requestid=3329186
11. Anti-GAPDH antibody, WB, Affinity, AF7021, 62u0922; http://www.affbiotech.cn/goods-6289-AF7021-GAPDH_Antibody.html
12. Anti-beta Tubulin antibody, WB, Invitrogen, MA5-11732, TBN06, (Tub2.5), TC253C541; https://www.thermofisher.cn/cn/zh/antibody/product/beta-Tubulin-Antibody-clone-TBN06-Tub-2-5-Monoclonal/MA5-11732
13. Anti-Histone H3 antibody, WB, Cell Signaling Technology, 4499, D1H2, 9; https://www.cellsignal.cn/products/primary-antibodies/histone-h3-d1h2-xp-rabbit-mab/4499?site-search-type=Products&N=4294956287&Ntt=4499&fromPage=plp&_requestid=3329246
14. Anti-Cyclin D1 antibody, IF, WB, Cell Signaling Technology, 55506, E3P5S, 3; https://www.cellsignal.cn/products/primary-antibodies/cyclin-d1-e3p5s-xp-rabbit-mab/55506?site-search-type=Products&N=4294956287&Ntt=55506&fromPage=plp&_requestid=3329273
15. Anti-GFP, WB, Cell Signaling Technology, 2956, D5.1, 1; https://www.cellsignal.cn/products/primary-antibodies/gfp-d5-1-rabbit-mab/2956?site-search-type=Products&N=4294956287&Ntt=2956&fromPage=plp&_requestid=3329312
16. Recombinant Anti-VEGFA antibody [EP1176Y]-C-terminal, IF, WB, Abcam, ab52917, EP1176Y; https://www.abcam.cn/vegfa-antibody-ep1176y-c-terminal-ab52917.html
17. Phospho-Smad1 (Ser463/465)/ Smad5 (Ser463/465)/ Smad9 (Ser465/467) (D5B10) Rabbit mAb, IF, WB, Cell Signaling Technology, 13820, Lot3; https://www.cellsignal.cn/products/primary-antibodies/phospho-smad1-ser463-465-smad5-ser463-465-smad9-ser465-467-d5b10-rabbit-mab/13820?site-search-type=Products&N=4294956287&Ntt=13820&fromPage=plp&_requestid=3329379
18. Anti-Fibronectin antibody, IF, WB, Abcam, ab2413, GR270934-1.
https://www.abcam.cn/fibronectin-antibody-ab2413.html

Eukaryotic cell lines

Policy information about cell lines

Cell line source(s)

MDCK II, human HEK293 cells, 3T3-L1, and mouse proximal tubule cells (TKPTS) were obtained from ATCC (USA) and cultured according to the distributor's recommendations.

Authentication	Cell lines have been authenticated by light microscopy but not by additional methods.
Mycoplasma contamination	Cell lines were tested monthly for mycoplasma contamination and all cell lines were negative.
Commonly misidentified lines (See ICLAC register)	No misidentified lines were used in this study.

Animals and other organisms

Policy information about studies involving animals; ARRIVE guidelines recommended for reporting animal research

Laboratory animals	The mouse strain we used was primarily C57BL/6J (B6) strain. Both male and female Cldn5 flox, Wif1 flox, Nphs2-cre mice were randomly used. The mouse ages ranged from 3 weeks to 48 weeks. Mice were housed in a standard environment which was characterized by 12 h light/dark cycle, 22-25°C and 40-60% humidity with free access to water and chow. Mice were usually sacrificed and analyzed a few weeks to a few months of age. We have established transgenic knockout mouse models for Cldn5 and Wif1 in pure C57BL/6 background. The 12-week-old male DB/M and DB/DB were obtained from Cavens Biogle (China). C57BL/6J (B6) mice were obtained from Vital River Laboratory (China). Other mouse models are available from the Jackson lab. 4-6 weeks male mice was used to induce the diabetic nephropathy by unilateral nephrectomy (UNX) combined with streptozotocin (STZ). Unilateral ureteral obstruction was performed on 8-10 weeks old male mice.
Wild animals	No wild animals were used in this study.
Field-collected samples	This study did not involve samples collected from the field.
Ethics oversight	All animal studies were approved by the Animal Ethics Committee of Binzhou Medical University and conducted in accordance with the National Institutes of Health Guide for the Care and Use of Laboratory Animals.

Note that full information on the approval of the study protocol must also be provided in the manuscript.
